# The local burden of disease during the first wave of the COVID-19 epidemic in England: estimation using different data sources from changing surveillance practices

**DOI:** 10.1186/s12889-022-13069-0

**Published:** 2022-04-11

**Authors:** Emily S. Nightingale, Sam Abbott, Timothy W. Russell, CMMID Covid-19 Working Group, Rachel Lowe, Graham F. Medley, Oliver J. Brady

**Affiliations:** 1 Department of Global Health and Development, London School of Hygiene and Tropical Medicine, London, UK; 2 Centre for Mathematical Modelling of Infectious Disease (CMMID), London School of Hygiene & Tropical Medicine, London, UK; 3 Department of Infectious Disease Epidemiology, London School of Hygiene and Tropical Medicine, London, UK; 4 Centre on Climate Change and Planetary Health, London School of Hygiene & Tropical Medicine, London, UK; 5 Barcelona Supercomputing Centre (BSC), Barcelona, Spain; 6 Catalan Institution for Research and Advanced Studies (ICREA), Barcelona, Spain

## Abstract

**Background:**

The COVID-19 epidemic has differentially impacted communities across England, with regional variation in rates of confirmed cases, hospitalisations and deaths. Measurement of this burden changed substantially over the first months, as surveillance was expanded to accommodate the escalating epidemic. Laboratory confirmation was initially restricted to clinical need (“pillar 1”) before expanding to community-wide symptomatics (“pillar 2”). This study aimed to ascertain whether inconsistent measurement of case data resulting from varying testing coverage could be reconciled by drawing inference from COVID-19-related deaths.

**Methods:**

We fit a Bayesian spatio-temporal model to weekly COVID-19-related deaths per local authority (LTLA) throughout the first wave (1 January 2020–30 June 2020), adjusting for the local epidemic timing and the age, deprivation and ethnic composition of its population. We combined predictions from this model with case data under community-wide, symptomatic testing and infection prevalence estimates from the ONS infection survey, to infer the likely trajectory of infections implied by the deaths in each LTLA.

**Results:**

A model including temporally- and spatially-correlated random effects was found to best accommodate the observed variation in COVID-19-related deaths, after accounting for local population characteristics. Predicted case counts under community-wide symptomatic testing suggest a total of 275,000–420,000 cases over the first wave - a median of over 100,000 additional to the total confirmed in practice under varying testing coverage. This translates to a peak incidence of around 200,000 total infections per week across England. The extent to which estimated total infections are reflected in confirmed case counts was found to vary substantially across LTLAs, ranging from 7% in Leicester to 96% in Gloucester with a median of 23%.

**Conclusions:**

Limitations in testing capacity biased the observed trajectory of COVID-19 infections throughout the first wave. Basing inference on COVID-19-related mortality and higher-coverage testing later in the time period, we could explore the extent of this bias more explicitly. Evidence points towards substantial under-representation of initial growth and peak magnitude of infections nationally, to which different parts of the country contribute unequally.

**Supplementary Information:**

The online version contains supplementary material available at 10.1186/s12889-022-13069-0.

## Introduction

The COVID-19 epidemic has impacted communities heterogeneously across England since evidence first emerged of local transmission in March 2020 [1]. Spatio-temporal patterns in transmission - driven, for example, by connectivity between regions, timing of initial exposure and impact of control measures - offer valuable insight into the development of the epidemic. However, such patterns are difficult to observe and interpret from raw reported case data alone, due to uneven vulnerabilities in the local population and changes in surveillance policy over time [2].

In particular, laboratory confirmation of cases was initially restricted to urgent clinical need of patients and healthcare staff (“pillar 1”) before being expanded to encompass all symptomatic cases in the wider community (“pillar 2”) from 18 May 2020 [3]. These data therefore reflect different subsets of total infections at different points of the epidemic. Deaths - in particular the broad class of COVID-19-*related* deaths, including both test-confirmed and clinically suspected cases (where the disease is considered to be the primary cause of death or a contributing factor) - can be considered more consistently recorded over time. We seek to exploit the biological link between the two sources of data to obtain a clearer picture of the burden of COVID-19 during the first wave, and to quantify the extent of under-ascertainment - by which we mean the gap between reported, confirmed cases and total infections - during scale up of testing.

Observed variation in the rate of COVID-19-related deaths can be considered a result of two spatially varying components: variation in incidence of infection and variation in fatality risk among those infected. Several individual-level characteristics have been highlighted as risk factors for COVID-19 case fatality - including age, deprivation and belonging to certain ethnic groups - all of which are themselves geographically clustered (Fig. S1). The influence of this on a population level is evident in local summaries of mortality rates in England and Wales [4], and will lead to substantial variation in the number of infections which give rise to observed deaths among the populations of different local areas. These factors should therefore be taken into account in order to understand how the relative number of deaths to infections varies over space and time. Changes in surveillance affect the probability of an infection being reported as a case and are therefore also important to account for when observing changes in the relative number of deaths to cases over time.

Previous studies have demonstrated several different methods for estimating the number of cases from reported deaths. Jombart et al. [5] offered an early attempt to infer symptomatic cases from the occurrence of a single death, concluding that there would have been in the region of several hundreds of cases by the time the first death was recorded. Russell et al. [6] proposed an approach based on published estimates of baseline case-fatality rates to estimate the proportion of unreported cases over time directly, at national and regional levels for a range of early-affected countries. Nicholson et al. [7] further discuss the impact of ascertainment bias in the UK’s surveillance systems and present an approach to quantify it through a joint analysis of targeted symptomatic and randomised testing data.

There have also been a number of studies exploring the spatial dynamics of COVID-19, within various country settings. Castro et al. [8] considered the timing of deaths and cases to understand the detected and undetected movement of the epidemic across Brazil. Cuadros et al. [9] explored differences in temporal trends in incidence rates between rural and urban counties in the US, but did not consider the proximity of counties in space. Amdaoud et al. [10] evaluated spatial autocorrelation statistics to analyse the early spread of COVID-19 across Western Europe, and explored how death rates related to demographic characteristics and measures of wealth, health care and social trust.

Other work exploring variation in mortality between local geographies of the UK has not accounted for the lack of independence between the units of interest, implicitly assuming that geographical regions can be considered independent after adjusting for a set of population covariates [11]. However, small and frequently zero counts in death data at a local level can limit precision of estimates when analysed independently. Sartorius et al. [12] do explicitly account for this dependence, adding a data-driven spatial structure in the form of correlated random effects within a mechanistic SEIR model, but fit to pillar one case counts only, assuming these represent a fitted proportion of total infections constrained between 5 and 40%, as informed by two systematic reviews of the asymptomatic proportion. This does not account for the proportion of individuals who are symptomatic but do not obtain a confirmed diagnosis.

This analysis aims to extend the concept of inferring infections and cases from deaths down to a local level while accounting for varying population characteristics, timing of first exposure and other unexplained sources of spatial correlation. With this approach we pursue a clearer understanding of the relative burden of disease across the country and how each locality contributes to the national picture.

## Materials and methods

### Data sources

Anonymised line lists of reported COVID-19-related deaths between 1 January and 30 June 2020 were provided by Public Health England (PHE). COVID-19-related deaths were considered to include those with COVID-19 recorded as an underlying cause, or where COVID-19 was mentioned as a contributing factor but not specified as cause of death. These two categories included a total of 52,560 reported deaths in England which occurred between 5 January and 30 June 2020, of which 39,332 had laboratory-confirmed infections and 13,228 non-confirmed but suspected. Counts were then aggregated by lower-tier local authority (LTLA), week of death (counted from Wednesday 1 January 2020) and 10-year age group. Aggregation by week was chosen in order to avoid excessive zero or low counts and potential day-of-week reporting effects, and to obtain a smoother representation of the epidemic curve. Records which did not have a LTLA provided (*n* = 74) were excluded. Local authority shapefiles and single-age population estimates were obtained from the Office for National Statistics (ONS) [13, 14] and matched to the aggregated death data. For descriptive purposes, the distributions of rates of deaths and confirmed cases across LTLAs are summarised by median and inter-quartile range (IQR).

LTLAs can be classified into one of four geographical categories: London borough (10.3% of total LTLAs), metropolitan district (11.5%), non-metropolitan district (60.3%) and unitary authority (17.9%). The former two categories capture the major urban areas of the country (including Birmingham, Liverpool, Manchester, Sheffield, Leeds and Newcastle) with high connectivity both nationally and internationally, while the latter capture predominantly rural areas and smaller towns or cities.

PCR-confirmed cases (i.e. COVID-19 infections identified through both pillar 1 and pillar 2 surveillance) were obtained from the same source and aggregated to the same spatial and temporal resolution. Finally, estimates of infection prevalence in England were obtained from the ONS COVID-19 infection survey pilot (15) which was initiated in May 2020. These are presented as an estimated percentage (plus 95% confidence interval based on the survey sample size) of the population who would test positive via PCR for COVID-19 during rolling fortnightly intervals.

### Case definitions

For the remainder of the paper, infections confirmed with a positive PCR test and recorded in official case data *prior* to the expansion of symptomatic community testing on 18 May 2020 will be referred to as *pre-P2 cases,* and infections confirmed *following* expansion of testing will be referred to as *post-P2 cases.* It is noted that, due to piloting of pillar 2 testing among high-risk groups, a proportion of pre-P2 cases will have been detected via the pillar 2 route. We conservatively define the surveillance policy change from the point at which pillar 2 was fully available to all symptomatic individuals - assuming that case data from this point most accurately reflect the increased coverage of the expanded system - and define the terminology according to this distinction. We will also introduce the concept of *predicted-P1+P2 cases*, meaning the predicted infections which would have been PCR-confirmed in the hypothetical scenario in which symptomatic community testing had been in place since the beginning of the epidemic (January 2020). These predicted-P1+P2 cases form a subset of total symptomatic cases, conditional on the additional criteria that the case must be both symptomatic and seek and obtain a confirmatory positive test result. Lateral flow devices were not introduced for asymptomatic testing until later in the year [3] and therefore are not considered here. All references to deaths imply *COVID-19-related deaths*, i.e., those where either PCR-confirmed or clinically diagnosed COVID-19 infection is recorded on the death certificate.

### Model structure

Bayesian mixed effects models for deaths per week and per LTLA were fitted using integrated nested Laplace approximation (INLA), implemented via the R package *R-INLA* [15, 16].

To facilitate comparison in observed deaths across local authorities with different population age distributions, age-adjusted expected deaths, *E*, were calculated for each LTLA to serve as an offset in models. Expected counts were based on age-specific weekly mortality rates averaged over the observed time period and over the country as a whole. See Additional file 1 for details. Weekly reported deaths in LTLA *i* were assumed to follow a negative binomial (NB) distribution, with log link function, offset by log (*E*_*i*_).

In addition to age, two population level characteristics were considered as risk factors for case fatality: level of deprivation and distribution of ethnicity. The Index of Multiple Deprivation (IMD) score is defined as a relative measure of deprivation between Lower Super Output Areas (LSOAs) and incorporates a range of social, economic and health factors [17]. LSOAs are defined such that each belongs to a unique LTLA, therefore IMD scores could be aggregated to the median across all LSOAs in each LTLA and categorised by quintiles. To account for the heterogeneous distribution of ethnicity across the country, the percentage of minority ethnic groups in each LTLA population (relative to white as the national majority) was calculated according to estimates from the most recent (2011) census of England and Wales (specifically table DC2101EW “Ethnic group by sex by age”, all persons and all age categories) [18]. The number of residents self-identifying as non-white was aggregated from a five-category classification (White, Mixed/multiple ethnic, Asian/Asian British, Black/African/Caribbean/Black British, and Other) and calculated as a proportion of the total LTLA population.

The temporal dependence in the data was modelled using a combination of random effects with random walk (RW) correlation structures [19]. A second-order random walk (RW2) on the number of weeks since the first observed death (the “epidemic” week) was intended to capture the shifted epidemic curve in each LA. Additionally, a first-order random walk (RW1) on calendar week was included to capture any overall deviations from these epidemic trends (potentially as a result of policy and behavioural change). As such, the number of deaths in any one LTLA during 1 week are a priori assumed to be correlated with the number of deaths across the prior 2 weeks. Models in which the second-order RW on epidemic week was fitted separately within each of the four geography categories were also considered.

Models without any specified spatial structure were compared to those with independent and identically-distributed (IID) random effects per LTLA, and with a combination of IID and structured, conditional auto-regressive effects (as described by Besag, York and Mollié [20], hereafter referred to as BYM), parameterised with a mixing parameter 𝜙 between the two [21]. The latter allowed assessment of the contribution of local spatial correlation to the fit of the model, relative to purely random (IID) variation.

Six models were fitted and compared:

(A) *Baseline* Observed deaths ~ log(E) (offset) + Overall epidemic trend (RW2) + calendar week trend (RW1) + covariates (IMD, % minority); no spatial structure.
(B) A + geography-dependent epidemic trends
(C) A + IID spatial structure.
(D) B + IID spatial structure.
(E) A + BYM spatial structure.
(F) B + BYM spatial structure.

The distributions of structured random effects (spatial and temporal) were fit with penalised complexity priors on the precision and BYM mixing parameters [22], and fixed effects fit with weakly-informative gaussian priors centred at zero. A more detailed specification of all models can be found in the Additional file 1.

All analyses were performed in R version 3.6.3 (2020-02-29). All code used to run these analyses have been made available at 10.5281/zenodo.5763664.

### Model comparison

Models were compared using the Widely Applicable Information Criterion (WAIC) [23] and log score [24]. Pearson residuals between fitted values and observed were averaged per LTLA and mapped as a visualisation of the spatial structure unexplained by each model. Posterior samples (*n* = 1000) were drawn to explore the uncertainty in predictions and aggregated over LTLAs to give total trajectories over time.

### Comparison to post-P2 cases

It was assumed that post-P2 cases (swabbed from 18 May 2020 onwards) were reflective of the higher coverage surveillance and less obscured by capacity constraints. A fixed lag of 1 week between date of swabbing and date of death was applied to infer *predicted-P1 + P2* cases from modelled deaths in the primary analysis, while a sensitivity analysis was conducted assuming two- and three-week lags. This choice was informed by the swab-death delay distribution observed in this dataset (median 6 days, IQR 8 days), while also considering an external report from the COVID Clinical Information Network (CO-CIN) [25] which suggested an overall longer and more varied distribution (median 13 days, IQR 14 days) between onset of symptoms and death. The possibility was considered that the lag between testing and death may have been shorter early in the epidemic, with cases predominantly being tested in a hospital setting when symptoms were already severe. However, the available data on swabbing and death dates did not suggest a difference between pre- and post-P2 cases (median 6 days pre-P2 and 7 days post-P2, with equal quartiles of 3–11 days), and therefore one fixed lag was assumed for the entire period. It was assumed that variation over this period of time in the ratio of post-P2 cases to deaths would be predominantly a result of varying completeness of observation of cases, rather than of a difference in underlying case-fatality risk.

The approach taken to infer predicted-P1 +P2 cases from reported deaths consisted of three steps. First, smoothed trajectories of deaths per week and per LTLA, corrected for spatial heterogeneity in case-fatality risk factors, were obtained from the fitted model (1000 posterior samples predicted at averaged covariate values with non-age-stratified population offset). An LTLA-level ratio of cases per covid-related death (post-P2 case per death ratio, CPDR) was then estimated for every week beyond 18 May 2020 and for each posterior sample, lagging the modelled death counts by 1 week (two and three weeks for the sensitivity analysis) and comparing to post-P2 cases. CPDRs were summarised over all post-P2 weeks to obtain a median and IQR for each LTLA, which were then used to scale up the posterior samples over the whole time period. This yielded an estimate of the magnitude of cases giving rise to those deaths, which would have been detected under expanded surveillance. The distributions across posterior samples are summarised into 1, 25, 75 and 99% quantiles - yielding 50 and 98% Credible Intervals (CrI) - for presentation.

### Inferring infection and rate of detection

The previous steps yield local trajectories of COVID-19 cases which would have been detected through combined hospital and community-based symptomatic testing, had such capacity been available throughout the first epidemic wave. However, post-P2 cases detected under expanded surveillance remain a subset of the total number of infections, which also include those that are asymptomatic or otherwise undetected. The ONS COVID-19 infection survey pilot [26] suggested that per fortnight between 27 April and 24 May 2020 around 0.25% of the population of England would have tested positive for COVID-19, with this percentage steadily decreasing to 0.03% by the beginning of July. To investigate the gap between total infection incidence and detected cases, these data were combined with post-P2 case counts over the same period to infer a rate of detection under expanded surveillance (see Additional file 1). This rate of detection was then applied to the entire trajectories of predicted-P+P2 cases to estimate the number of infections represented by those detected cases. Observed pre- and post-P2 counts could then be compared to these estimated infections to infer the percentage of infections detected over time and within each LTLA.

## Results

**Fig. 1:**
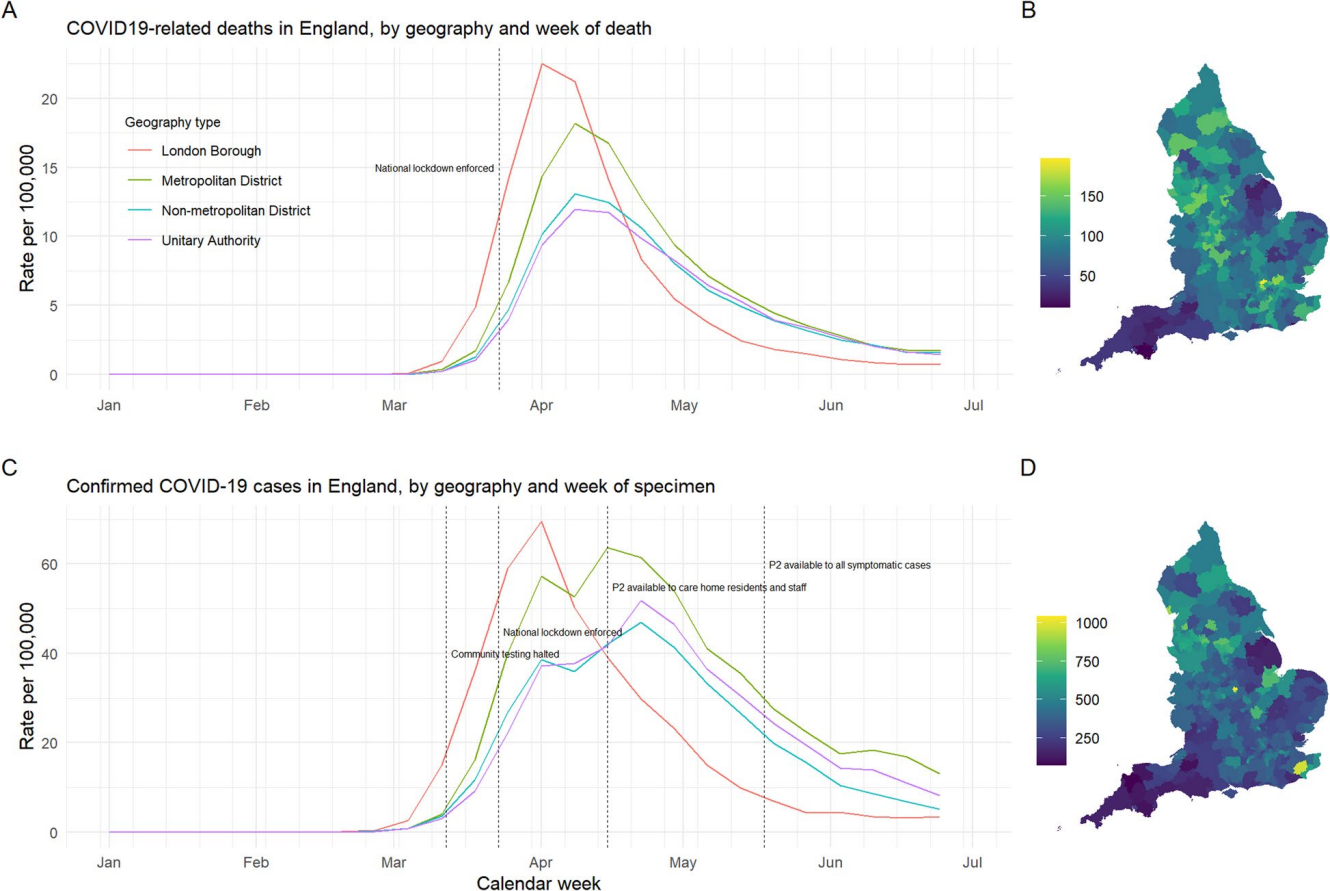
Rates of COVID-19-related deaths and confirmed cases in England, by geography and week of death, and by lower-tier local authority (LTLA). **(A, B):** Weekly rates per 100,000 population of COVID-19-related deaths and confirmed cases, respectively, by geography type. Trajectories of reported deaths follow a smooth epidemic curve while the peak in case counts appears to be truncated across geographies outside of the early-affected London region, potentially as a result of national lockdown measures but also of testing constraints. Dashed vertical lines mark dates of significant policy changes with respect to confirmatory testing of suspect cases. **(C, D):** The same data instead presented as total rates per 100,000 per LTLA, across the entire first wave (1 January 2020 to 30 June 2020). Time periods are set according to the date of specimen and date of death, respectively

A summary of the observed incidence of covid-related deaths and pre−/post-P2 confirmed cases is shown in Fig. 1. Over time, the early exposure of London is clear in both deaths and confirmed cases, with the two epidemic curves following a similar shape and peaking prior to the other geographies. Outside of London, confirmed case counts appear to be truncated between the end of March and the end of April, approximately coinciding with the implementation of the national lockdown on 23 March 2020.

Overall, COVID-19-related mortality rates ranged from 10 per 100,000 in South Hampshire to 196 per 100,000 in Hertsmere (median [IQR]: 90.6 [71.4, 112.1]). Cumulative incidence of confirmed cases was more varied between LTLAs, ranging from 71 per 100,000 in Torridge in North Devon, to 1040 per 100,000 in the East Midlands city of Leicester (median [IQR]: 379.2 [298.3, 491.5]). Supplementary Fig. S1 illustrates the substantial variation in the population characteristics assumed to contribute to case-fatality risk across the country.

### Model selection

By comparison of information criteria (WAIC) and cross-validated log score, it is clear that adjustment for epidemic timing and the specified fatality risk covariates (model A) were insufficient alone to explain the spatial distribution of deaths across England. Out of the six candidate models, the BYM spatial model and temporal trends specific to the geography of the LTLA was selected as offering the lowest WAIC and best cross-validated fit (model F) (Table 1). Models with unstructured, IID random effects per LTLA performed comparably to the BYM model and the overall magnitude of error appeared to be reduced, but spatial structure in the residuals was still evident (Supplementary Fig. S2).

**Table 1:** Overall model comparison by WAIC and log score

Model	WAIC	Log score	Diff WAIC	Diff log score
**B + BYM spatial**	**24,750**	**2.601**	**–**	**–**
**B** + IID spatial	24,801	2.606	51	0.005
**A** + BYM spatial	25,602	2.690	851	0.089
**A** + IID spatial	25,665	2.697	914	0.096
Geog-specific temporal (**B**)	26,344	2.768	1593	0.167
Temporal only (**A**)	26,865	2.823	2115	0.222

### Final model

The final model suggested strong associations between weekly rates of COVID-19-related deaths in a LTLA, quintiles of deprivation score and proportion of minority ethnicities in the population (RR = 1.27 with 95% CrI [1.10–1.47] between the 1st and 4th quintiles of IMD; RR = 1.01 [1.006–1.015] per percentage increase in minority ethnic population), after adjusting for the size and age distribution of the local population (Supplementary Table S2). Despite a clear monotonic trend through the first four quintiles of deprivation score, the difference between the 1st and 5th (most deprived) quintiles dropped slightly and was estimated with a wider CrI (RR = 1.21 [0.97, 1.49]), perhaps due to the smaller number of LTLAs which fall into this category. Differences in the shape of the epidemic between each geography type were best captured by four separately fitted trends as opposed to one overall trend, and residual heterogeneity between LTLAs (i.e., not captured by covariates) was explained by a combination of spatial correlation and random noise.

**Fig. 2:**
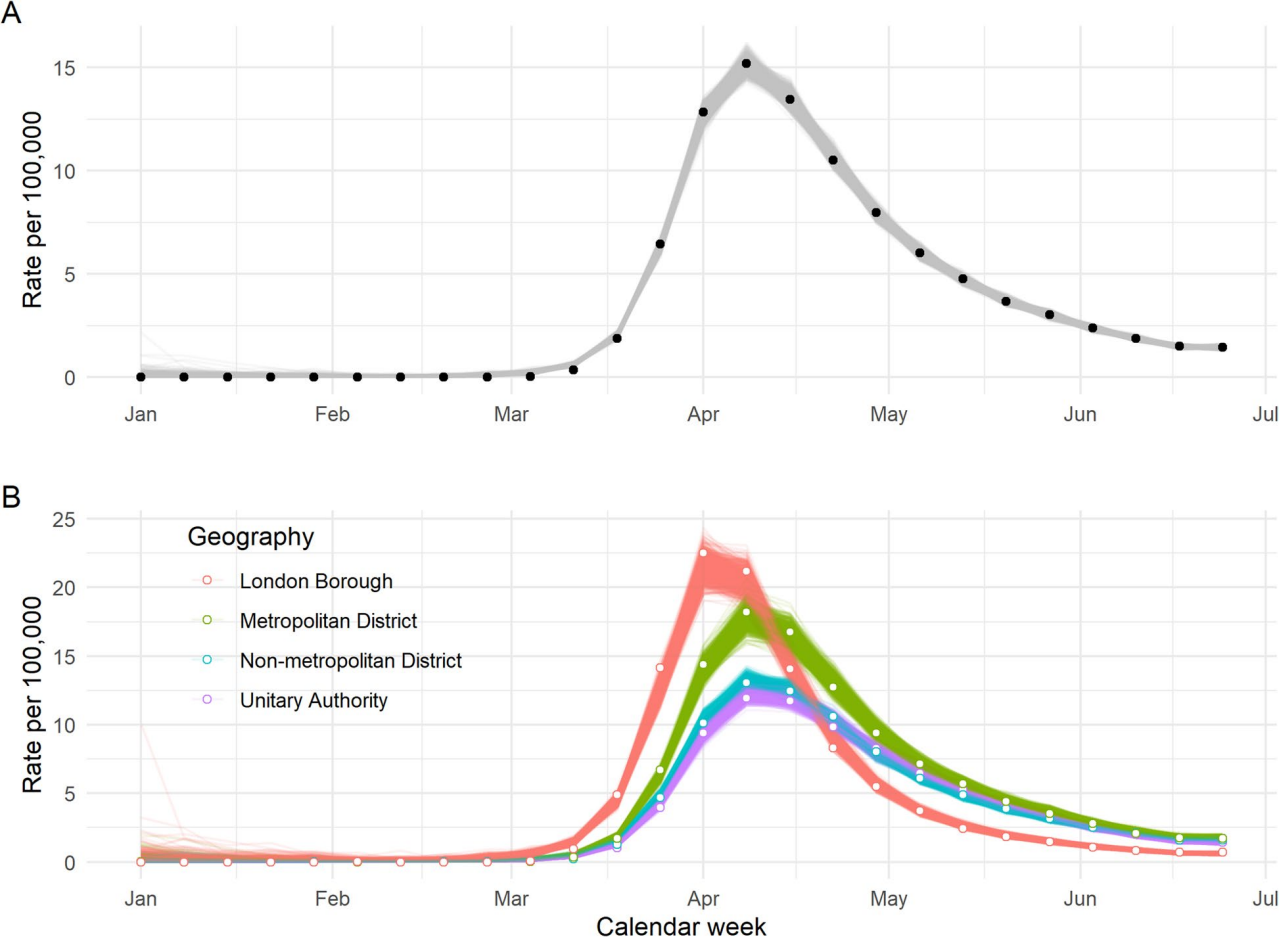
Final model fit (1000 posterior samples) over time, as a national total and by geography type. The final model describes observed weekly COVID-19-related deaths per LTLA in terms of the size, age, ethnicity and deprivation level of the population, temporal trend and spatial correlation between neighbouring LTLAs. Observed rates of covid-related death per 100,000 population are shown in black (A) and white (B). Each grey/coloured line represents one sampled trajectory from the fitted model, and variation between these reflects uncertainty in the fit

Posterior samples drawn from the selected model illustrated a close fit to the epidemic trajectories overall and within each specific geography (Fig. 2). Fits for a random sample of individual LTLAs are illustrated in Supplementary Fig. S3.

The fitted posterior for the BYM mixing parameter, Φ, implies that at least 86% (posterior mean 95, 95% CrI: [86–99%]) of the residual spatial variation (accounting for the specified covariates and temporal trends) could be explained by correlation between neighbouring LTLAs as opposed to random noise. This suggests that there is correlation in observed mortality in neighbouring areas which is not explained by similarities in the size, age distribution, ethnic composition or deprivation level of their populations. A decomposition of the fitted spatial random effects for each LTLA is illustrated in Supplementary Fig. S4.

### Comparison to post-P2 cases

Prior to the expansion of pillar 2 surveillance, the median CPDR per LTLA was 4.1 confirmed cases per covid-related death (IQR [3.4,5.0]). From 18 May 2020 onwards, this increased to a median of 5.2 with more variation between LTLAs (IQR [3.3,8.6]). Further detail of the spatial heterogeneity in CPDR across the country is illustrated in Supplementary Fig. S5.

**Fig. 3:**
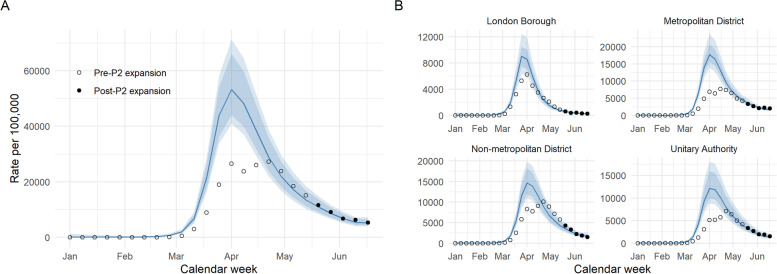
Predicted-P1+P2 cases, according to lagged and scaled-up predictions from the selected model for COVID-19-related deaths, in total (A and aggregated by geography type (B). 50–98% credible intervals are shown by the blue shaded areas. Observed totals of confirmed cases per week are indicated by black points - unfilled prior to P2-expansion and filled post-P2 expansion. Predicted-P1+P2 cases suggest the potential shape and magnitude of the first wave peak if community symptomatic testing (pillar 2) - in addition to hospital-based testing (pillar 1) - had been available from the beginning of the epidemic

Figure 3 illustrates the trajectories of predicted-P1+P2 cases inferred from the model-predicted deaths per LTLA, aggregated overall and by geography. Although the more comprehensive surveillance was assumed to be in place by mid-May, the trajectories of inferred and actual cases appear similar from the end of April to early May, when testing was accessible to care home residents and staff, over 65s and key workers [19]. Overall, the reconstructed epidemic curve of predicted-P1+P2 cases yields a median of over 100,000 additional cases - an increase of 45% - over the course of the first wave (Table 2).

**Table 2:** Summary of observed and predicted-P1+P2 case counts over the first wave, nationally and by geography

	Observed, test- confirmed cases (***up to week starting 2020-06-17***)	Predicted (***median [98% CrI]***)	Percentage difference
England total	231,817	335,083 [275,482 - 418,847]	44.5 [18.8–80.7]
London Borough	33,399	43,664 [35,881 - 51,337]	30.7 [7.4–53.7]
Metropolitan District	64,007	109,717 [95,734 - 129,216]	71.4 [49.6–101.9]
Non-metropolitan District	79,441	97,786 [78,764 - 122,095]	23.1 [−0.9–53.7]
Unitary Authority	54,970	83,723 [67,673 - 110,968]	52.3 [23.1–101.9]

The four geography types contribute unevenly to this difference. In London, the reconstructed counts suggest a relative under-representation of the peak incidence in observed confirmed cases, which narrows relatively rapidly from April onwards as numbers decline and testing capacity increases. The implied under-ascertainment in London is of a much smaller magnitude than the other three geographies; in particular for metropolitan districts and unitary authorities, results suggest that, at the height of the epidemic, confirmed cases potentially constituted less than half of the symptomatic cases which would have been detected under the expanded system. For the predominantly rural non-metropolitan districts the difference at the peak is less substantial, though still greater than that of London. From late-April, total confirmed case incidence across these LTLAs actually exceeds the reconstructed counts, by a small margin which diminishes towards the beginning of the summer (see Fig. 3A).

**Fig. 4:**
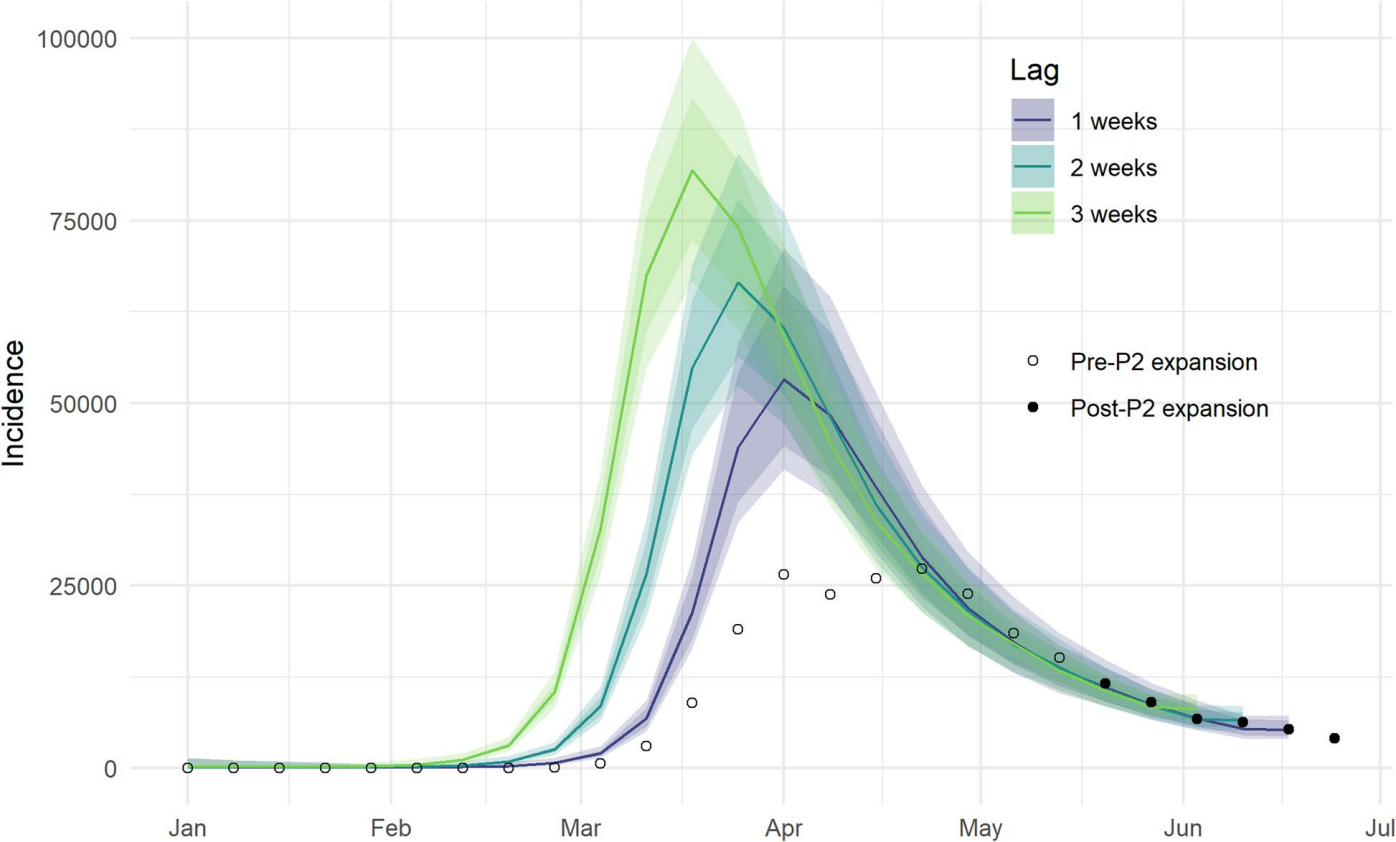
Comparison of predicted-P1+P2 cases assuming one-, two- and three-week lags between date of swabbing and date of death. Shaded intervals represent 50–98% credible intervals

Assuming a longer two-week lag between testing and death yields a much larger difference of 86%, and for 3 weeks this increases further to 135%. A comparison of reconstructed national totals based on three different lags is included in Supplementary Table S3 and illustrated in Fig. 4. It is clearly shown that assuming a longer lag between case confirmation and death yields a higher and earlier peak in the reconstructed trajectory of cases.

### Estimation of total infections

**Fig. 5:**
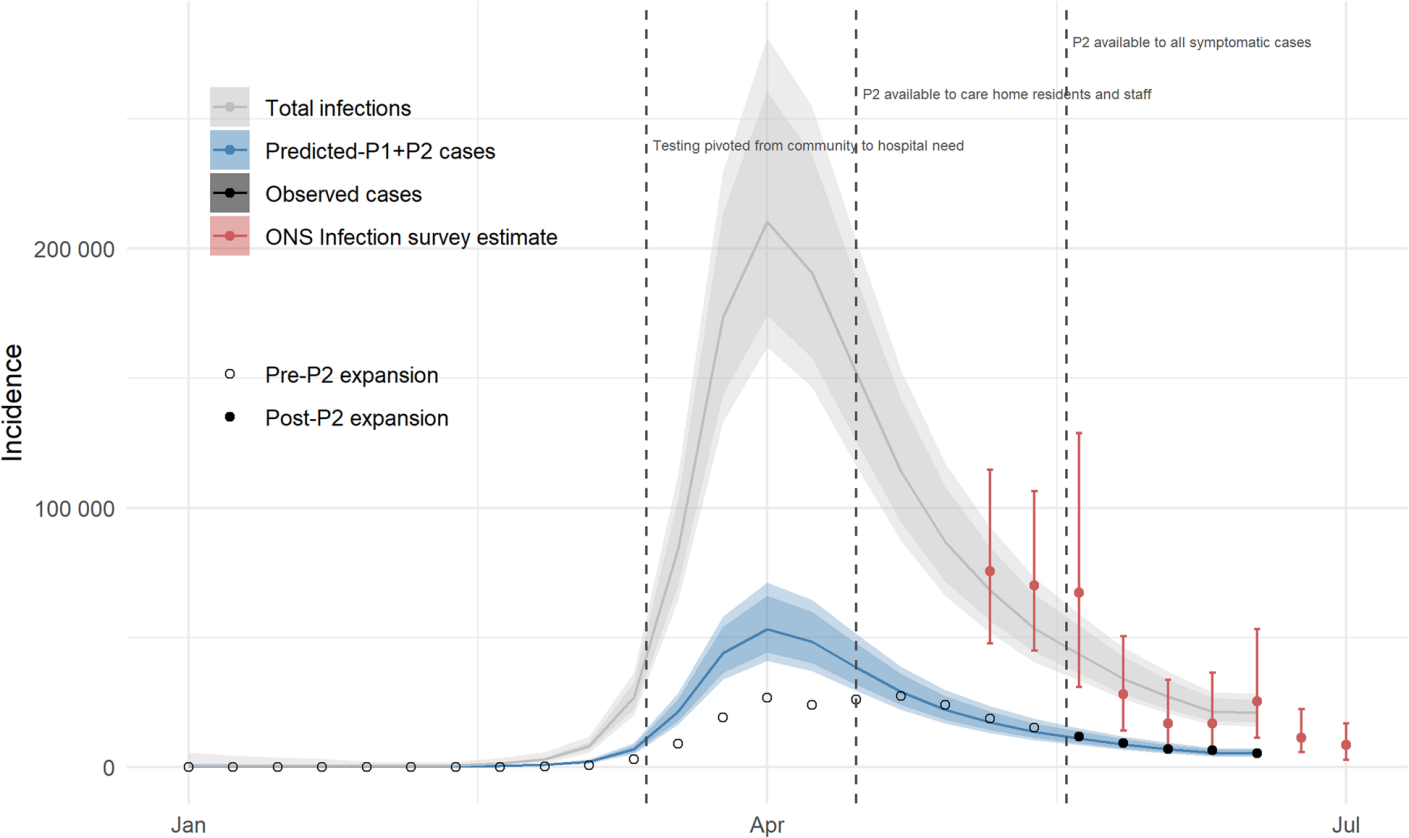
Estimated weekly incidence of infections in England (grey), inferred from predicted-P1+P2 cases (blue) and an assumed detection rate of 25% under expanded surveillance. Rate of detection is estimated by comparison of incidence estimates from the ONS infection survey (shown in red) and observed case counts (shown in black) between weeks starting 18 May to 15 June 2020. This rate is then applied to predicted-P1+P2 cases to obtain the estimated trajectory of total infections

Figure 5 illustrates the estimated national incidence of infection according to the ONS infection survey pilot, alongside total observed and reconstructed cases across the country. Comparison of observed cases from weeks starting 18 May to 15 June 2020 with these estimated total infections suggested an overall rate of detection of 25% (95% CI propagated from infection prevalence estimates: 13–58%). The total wave of infections over the entire period implied by this rate of detection is indicated by the grey curve.

This yields a cumulative total of 1.3 million infections (98% CrI 1.04 to 1.74 million) throughout the first wave, of which the observed confirmed cases (*n* = 231,817) constitute 17.5% (98% CrI 13.3 to 22.3%).

**Fig. 6:**
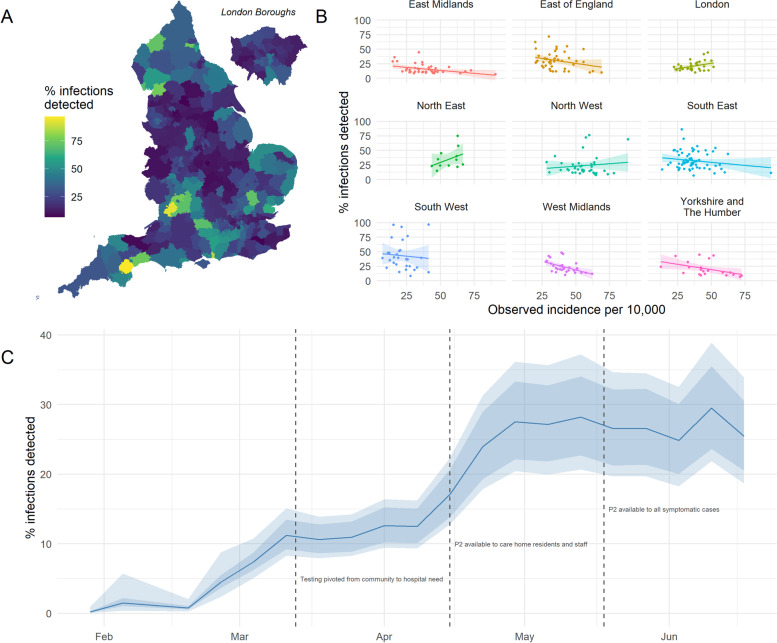
Estimated percentage of total infections represented in observed case counts, per LTLA (panels **A** and **B**) and per week (panel **C**), between 2020 and 01-01 and 2020-06-17. LTLAs of Gloucester and Teignbridge stand out as having the highest percentage of detected infections, with estimates of over 96%. Panel **B** illustrates the same estimates in panel **A** but grouped by region, against the total observed incidence per 100,000. Total infections over the time period are estimated based on the predicted-P1+P2 cases and an assumed infection detection rate of 25% under expanded surveillance. All panels reflect median predictions over 1000 posterior samples, with panel **C** additionally showing 50–98% credible intervals

Within each LTLA, cumulative incidence of confirmed cases constituted a median of 23% of estimate total infections (Fig. 6A). The highest rates of detection were found in Gloucester and Teignbridge in the south-west, both with estimates of over 96% (98% CrIs [87, 110%] and [81, 121%], respectively), while less than 7% detection was estimated in Leicester, Tunbridge Wells and Bradford (98% CrIs [3, 11%], [6, 15%] and [4, 10%], respectively). See Supplementary Fig. S6 for predicted trajectories in these LTLAs. Figure 6B presents the estimated detection rate for each LTLA compared to the total observed incidence, grouped by region. In most regions, greater observed incidence coincides with poorer detection of total infections. However, in London and the North, the trend leans more into the opposite direction. Supplementary Table S4 reports cumulative estimates of total infections nationally, by geography type and by region. By week, the level of under-ascertainment decreases in magnitude from February to April and settles between 25 and 30% from late-April to June (Fig. 6C).

The final predicted-P1+P2 and total infections for the entire time series in each LTLA are included in Additional file 2.

## Discussion

This analysis has demonstrated that it is possible to generate plausible case burden estimates from COVID-19-related death data and, in doing so, investigate the impact of changes in surveillance practices over the first months of the epidemic.

The model development process highlights a clear spatial structure to the incidence of covid-related deaths at a sub-national level, which is not explained by variation in the timing of initial exposure (epidemic week), or well-documented risk factors of COVID-19 death (age, deprivation and ethnic distribution in the local population). Similarities in risk between neighbouring LTLAs can be an important factor to consider for the design of local mitigation strategies, particularly in response to the detection of new variants.

Assuming that the epidemic curve of deaths represents a specific subset of total symptomatic - i.e., detectable under Pillar 1 and 2 testing strategies - infections, this analysis suggests that over 100,000 additional cases may have been counted across the country in the absence of the initial constraints on testing capacity. The uncertainty around this estimate is relatively broad (98% CrI [44,000 - 250,000]), predominantly as a result of uncertainty in the case-per-death ratio used to translate between the two measures. The increased heterogeneity between LTLAs in estimated CPDR following pillar 2 expansion may to some extent be attributed to much lower counts of both cases and deaths as the epidemic waned, and the occurrence of local outbreaks. Overall, we estimate around four post-P2 confirmed cases per covid-related death across all LTLAs, or equivalently a rate of 0.25 deaths per case. This is higher than estimates of the case-fatality rate i.e. the rate of deaths among confirmed cases, due to our broader definition of covid-*related* deaths as opposed to deaths directly attributed to COVID-19 *among* confirmed cases.

Cases ascertained, even under the expanded system, remain a subset of total infections. The case estimates obtained here were therefore combined with estimates of infection incidence from the ONS’s pilot survey in order to explore the rate of detection over time and between LTLAs. This investigation suggested that, following the roll-out of symptomatic community testing, around a quarter of infections in England were being detected - a value consistent with estimates obtained by Colman et al. [27] for the period of June to November 2020 - compared to only around 10% during the first months of the pandemic.

The extent of this under-ascertainment was found to vary not only over time alongside the expansion of testing capacity, but also between LTLAs. Comparing the final model fit to the observed deaths suggested relatively little deviation of each LTLA from the fitted geography-specific trends, yet the reconstructed infections differ from observed test positives with much more variation between LTLAs. Greater observed incidence rates appeared to coincide with poorer detection of total infections - perhaps reflecting the impact of reaching testing capacity - yet this trend was found to be inconsistent between regions. This demonstrates the sporadic nature of case observation over time and space and highlights certain areas of the country in which surveillance was perhaps more strongly impacted by testing constraints. These differences may in part be attributed to local variation in the relationship between cases and deaths which isn’t sufficiently captured by the assumed case-fatality covariates. We therefore advise that LTLA-specific estimates should be interpreted with consideration of the local context.

Early projections based on critical care admissions by Jit et al. [28] suggested an incidence of over 8000 infections per day in the UK by mid-March 2020. Assuming detection of 25% of infections under hospital and community symptomatic testing, from this study we estimate a total of around 111,000 infections during the two middle weeks of March, equating to an average of just under 8000 per day in England alone. On the other hand, via a mechanistic modelling approach, another study estimated daily total infections in the UK to have reached in the region of several hundred thousand by late-March [29]**.** Genomic analysis suggests that importations into the UK alone peaked mid-March with up to 1000 per day [30].

Russell et al. [6] took a data-driven approach in estimating that the peak incidence of *symptomatic* infections across the UK had occurred by mid-April 2020 with a magnitude of around 100,000 per day, and concluded that during March only 3–10% of such cases were being detected. This suggests a substantially higher peak than our estimate for England alone of just over 200,000 *total* infections per week - on average 28,000 per day - even accounting for the distribution of population between the constituent countries. Our estimates suggest that the percentage of these total infections reflected in confirmed case counts varied from around 7% at the start of March to 11% at the end, slightly higher than the detection rate Russell et al. estimated among symptomatic infections. Overall, estimates of infection incidence appear to be variable across studies, at least in part due differences in case definitions and aggregation over space and time.

### Limitations

The interpretation of these findings depends on several key assumptions, most importantly that variability over time in the ratio of confirmed cases to COVID-19-related deaths is predominantly the result of varying accessibility of testing. It is however plausible that fatality risk would have varied over time, potentially increasing towards the peak of the wave due to strain on hospitals forcing re-prioritisation of care or decreasing later on as treatment options improved. Also, it was assumed that variability in the delay from swabbing to death on the individual level would be diluted by aggregation, hence a fixed-value lag (with its influence explored in sensitivity analysis) would suffice. Summarising observed delays between swabbing and death within the available data gave no reason to suggest a difference between the time periods pre- and post-pillar 2 expansion, therefore the same fixed lag was assumed throughout the epidemic wave. A more exact approach, however, would have been to incorporate the full distribution of swab-death delays and redistribute the observed deaths in time according to an imputed point of detection.

Only three broad characteristics were considered as case-fatality risk factors, which essentially serve as proxy measures for complex combinations of underlying comorbidities and health indicators across the population. Dichotomising self-identified ethnicity in a population into “majority” and “minority” groups is crude, given that risk has been found to differ between ethnic groups in different ways [31]. We implicitly assume that these estimates from the national census are representative of the population. Several studies report case-fatality risk as being overall higher among biological males [32–34], yet there is also debate as to how the effect may interact with other key risk factors such as age and deprivation [35]. Here it was found that the ratio of males to females varied only marginally between LTLA populations and was uninformative for the observed mortality rate.

In individual level analysis, comorbidities such as cardiovascular disease, diabetes, and cancer were shown to have an association with mortality after adjusting for both ethnicity and deprivation level [36]. The local prevalence of such conditions is however a component in the calculation of the deprivation score used here. There are likely nuances and complex interactions between granular risk factors for mortality [37, 38] which are not yet understood in sufficient detail to be explicitly defined in such a model. The measures used here are therefore intended to capture high-level differences, and further work exploring additional covariates associated with mortality on both an individual and environmental/contextual level might improve population-level risk estimates.

Finally, this approach does not account for the dynamics of transmission in and around long-term residential care facilities during the early months of the pandemic, within which many deaths during the first wave occurred [39]. The nature of infections in these settings - with respect to mortality, testing and management - is different to that which occurred in the wider community, yet both care home and community deaths were treated equally in this analysis. For LTLAs with a particularly large care home population, the estimated case-per-death ratio may be higher than it would have been excluding these particularly vulnerable individuals. However, adjusting for the age distribution of the LTLA population in the underlying deaths model should at least in part attenuate this source of variation. This study aimed to explore broad, population-level patterns in incidence of deaths and detection of cases, whereas characterising the contribution of incidence within residential care settings would require a more fine-scaled, context-specific analysis. There was further substantial transmission within healthcare settings which we have not included separately [40].

## Conclusions

Effective and efficient control of an infectious disease epidemic relies on appropriate quantification of risk at a local level from available surveillance data. However, there are many reasons for which such data may not be equally representative of disease burden across different regions and populations. In the case of the COVID-19 epidemic in England, it is known that limitations in testing capacity distorted the observed trajectory of cases during the first wave. In this analysis, by combining more consistently reported data on deaths and more representative case data from later in the epidemic, it was possible to reconstruct a plausible trajectory of symptomatic cases which could have been detected in the absence of the early testing constraints, and further to infer the total number of infections these reported cases would represent. This facilitated a comparison between the two testing policies and highlighted heterogeneity in case ascertainment across different regions of the country.

The burden of disease and impact of the response to this pandemic will be evaluated in detail for years to come. Considering how changes in surveillance policy can obscure the spread of an epidemic - using methods such as those demonstrated here - will be essential, in particular for understanding the consequences of the country’s initial level of pandemic preparedness.

## Supplementary Information

**Additional file 1.** Supplementary Materials.

**Additional file 2.**  Final predicted-P1+P2 and total infections for the entire time series in each LTLA.

## Data Availability

The datasets supporting the conclusions of this article are available at 10.5281/zenodo.5763664.

## Abbreviations

BYM: Besag York Mollié
CPDR: Cases Per Death Ratio
CO-CIN: COVID Clinical Information Network
CrI: Credible Interval
IID: Independent and Identically Distributed
IMD: Index of Multiple Deprivation
INLA: Integrated Nested Laplace Approximation
IQR: Inter-Quartile Range
LSOA: Lower Super Output Area
LTLA: Lower Tier Local Authority
ONS: Office for National Statistics
PCR: Polymerase chain reaction
RW: Random Walk
WAIC: Widely Applicable Information Criterion

## References

[1] Holden B, Quinney A, Padfield S, Morton W, Coles S, Manley P, et al. COVID-19: public health management of the first two confirmed cases identified in the UK. Epidemiol Infect. 2020;148 Available from: https://www.ncbi.nlm.nih.gov/pmc/articles/PMC7484301/. 10.1017/S0950268820001922 PMC7484301 32854791

[2] Sherratt K, Abbott S, Meakin SR, Hellewell J, Munday JD, Bosse N, et al. Exploring surveillance data biases when estimating the reproduction number: with insights into subpopulation transmission of COVID-19 in England. Philos Trans R Soc B Biol Sci. 2021;376(1829):20200283. 10.1098/rstb.2020.0283 PMC8165604 34053260

[3] Coronavirus (COVID-19): scaling up testing programmes [Internet]. GOV.UK. Available from: https://www.gov.uk/government/publications/coronavirus-covid-19-scaling-up-testing-programmes. Accessed 14 May 2021.

[4] Deaths involving COVID-19 by local area and socioeconomic deprivation - Office for National Statistics [Internet]. Available from: https://www.ons.gov.uk/peoplepopulationandcommunity/birthsdeathsandmarriages/deaths/bulletins/deathsinvolvingcovid19bylocalareasanddeprivation/deathsoccurringbetween1marchand17april. Accessed 17 Mar 2021.

[5] Jombart T, van Zandvoort K, Russell TW, Jarvis CI, Gimma A, Abbott S (2020). Inferring the number of COVID-19 cases from recently reported deaths. Wellcome Open Res..

[6] Russell TW, Golding N, Hellewell J, Abbott S, Wright L, Pearson CAB (2020). Reconstructing the early global dynamics of under-ascertained COVID-19 cases and infections. BMC Med..

[7] Nicholson G, Lehmann B, Padellini T, Pouwels KB, Jersakova R, Lomax J, et al. Improving local prevalence estimates of SARS-CoV-2 infections using a causal debiasing framework. Nat Microbiol. 2022;7(1):97–107. 10.1038/s41564-021-01029-0 PMC8727294 34972825

[8] Castro MC, Kim S, Barberia L, Ribeiro AF, Gurzenda S, Ribeiro KB, et al. Spatiotemporal pattern of COVID-19 spread in Brazil. Science. 2021 Apr 14;372(6544):821–6. 10.1126/science.abh1558 33853971

[9] Cuadros DF, Branscum AJ, Mukandavire Z, Miller FD, MacKinnon N (2021). Dynamics of the COVID-19 epidemic in urban and rural areas in the United States. Ann Epidemiol..

[10] Amdaoud M, Arcuri G, Levratto N (2021). Are regions equal in adversity? A spatial analysis of spread and dynamics of COVID-19 in Europe. Eur J Health Econ..

[11] Verhagen MD, Brazel DM, Dowd JB, Kashnitsky I, Mills MC (2020). Forecasting spatial, socioeconomic and demographic variation in COVID-19 health care demand in England and Wales. BMC Med..

[12] Sartorius B, Lawson AB, Pullan RL (2021). Modelling and predicting the spatio-temporal spread of COVID-19, associated deaths and impact of key risk factors in England. Sci Rep..

[13] Local Authority Districts (April 2019) Names and Codes in the United Kingdom [Internet]. Available from: https://geoportal.statistics.gov.uk/datasets/c3ddcd23a15c4d7985d8b36f1344b1db_0. Accessed 17 Mar 2021.

[14] Population estimates for the UK, England and Wales, Scotland and Northern Ireland - Office for National Statistics [Internet]. Available from: https://www.ons.gov.uk/peoplepopulationandcommunity/populationandmigration/populationestimates/bulletins/annualmidyearpopulationestimates/mid2019estimates. Accessed 17 Mar 2021.

[15] Rue H, Martino S, Chopin N (2009). Approximate Bayesian inference for latent Gaussian models by using integrated nested Laplace approximations. J R Stat Soc Ser B Stat Methodol..

[16] Martins TG, Simpson D, Lindgren F, Rue H (2013). Bayesian computing with INLA: New features. Comput Stat Data Anal..

[17] English indices of deprivation 2019 [Internet]. GOV.UK. Available from: https://www.gov.uk/government/statistics/english-indices-of-deprivation-2019. Accessed 1 Feb 2021.

[18] DC2101EW (Ethnic group by sex by age) - Nomis - Official Labour Market Statistics [Internet]. Available from: https://www.nomisweb.co.uk/census/2011/dc2101ew. Accessed 1 Feb 2021.

[19] Codling EA, Plank MJ, Benhamou S (2008). Random walk models in biology. J R Soc Interface..

[20] Besag J, York J, Mollié A (1991). Bayesian image restoration, with two applications in spatial statistics. Ann Inst Stat Math..

[21] Riebler A, Sørbye SH, Simpson D, Rue H. An intuitive Bayesian spatial model for disease mapping that accounts for scaling. Stat Methods Med Res. 2016;25(4):1145–65. 10.1177/0962280216660421 27566770

[22] Simpson D, Rue H, Riebler A, Martins TG, Sørbye SH (2017). Penalising Model Component Complexity: A Principled, Practical Approach to Constructing Priors. Stat Sci..

[23] Watanabe S (2010). Asymptotic Equivalence of Bayes Cross Validation and Widely Applicable Information Criterion in Singular Learning Theory. J Mach Learn Res..

[24] Gneiting T, Aftery AER (2007). Strictly Proper Scoring Rules , Prediction , and Estimation. J Am Stat Assoc..

[25] CO-CIN: COVID-19 - Time from symptom onset until death in UK hospitalised patients, 7 October 2020 [Internet]. GOV.UK. Available from: https://www.gov.uk/government/publications/co-cin-covid-19-time-from-symptom-onset-until-death-in-uk-hospitalised-patients-7-october-2020. Accessed 20 Nov 2020.

[26] Coronavirus (COVID-19) Infection Survey: England - Office for National Statistics [Internet]. Available from: https://www.ons.gov.uk/peoplepopulationandcommunity/healthandsocialcare/conditionsanddiseases/datasets/coronaviruscovid19infectionsurveydata. Accessed 14 Oct 2021.

[27] Colman E, Enright J, Puspitarani G, Kao R. Estimating the proportion of SARS-CoV-2 infections reported through diagnostic testing. medRxiv. 2021. 10.1101/2021.02.09.21251411.

[28] Jit M, Jombart T, Nightingale ES, Endo A, Abbott S, Group LC for MM of IDC-19 W (2020). Estimating number of cases and spread of coronavirus disease (COVID-19) using critical care admissions, United Kingdom, February to March 2020. Eurosurveillance.

[29] Flaxman S, Mishra S, Gandy A, Unwin HJT, Mellan TA, Coupland H (2020). Estimating the effects of non-pharmaceutical interventions on COVID-19 in Europe. Nature..

[30] du Plessis L, McCrone JT, Zarebski AE, Hill V, Ruis C, Gutierrez B, et al. Establishment and lineage dynamics of the SARS-CoV-2 epidemic in the UK. Science. 2021;371(6530):708–12. 10.1126/science.abf2946 PMC7877493 33419936

[31] Collaborative TO, Mathur R, Rentsch CT, Morton CE, Hulme WJ, Schultze A, et al. Ethnic differences in COVID-19 infection, hospitalisation, and mortality: an OpenSAFELY analysis of 17 million adults in England. medRxiv. 2020; 10.1016/S0140-6736(21)00634-6.

[32] Peckham H, de Gruijter NM, Raine C, Radziszewska A, Ciurtin C, Wedderburn LR (2020). Male sex identified by global COVID-19 meta-analysis as a risk factor for death and ITU admission. Nat Commun..

[33] Bienvenu LA, Noonan J, Wang X, Peter K. Higher mortality of COVID-19 in males: sex differences in immune response and cardiovascular comorbidities. Cardiovasc Res. 2020; 10.1093/cvr/cvaa284. 10.1093/cvr/cvaa284 PMC7665363 33063089

[34] Gebhard C, Regitz-Zagrosek V, Neuhauser HK, Morgan R, Klein SL (2020). Impact of sex and gender on COVID-19 outcomes in Europe. Biol Sex Differ..

[35] Dehingia N, Raj A (2021). Sex differences in COVID-19 case fatality: do we know enough?. Lancet Glob Health..

[36] Williamson EJ, Walker AJ, Bhaskaran K, Bacon S, Bates C, Morton CE (2020). Factors associated with COVID-19-related death using OpenSAFELY. Nature..

[37] Lassale C, Gaye B, Hamer M, Gale CR, Batty GD (2020). Ethnic disparities in hospitalisation for COVID-19 in England: The role of socioeconomic factors, mental health, and inflammatory and pro-inflammatory factors in a community-based cohort study. Brain Behav Immun..

[38] Townsend MJ, Kyle TK, Stanford FC (2020). Outcomes of COVID-19: disparities in obesity and by ethnicity/race. Int J Obes..

[39] Gordon AL, Goodman C, Achterberg W, Barker RO, Burns E, Hanratty B (2020). Commentary: COVID in care homes—challenges and dilemmas in healthcare delivery. Age Ageing..

[40] Knight G, Pham TM, Stimson J, Funk S, Jafari Y, Pople D, et al. The contribution of hospital-acquired infections to the COVID-19 epidemic in England in the first half of 2020. Res Sq. 2022. 10.21203/rs.3.rs-1140332/v1. 10.1186/s12879-022-07490-4 PMC9206097 35717168

