# Supplementary material for: The local burden of disease during the first wave of the COVID-19 epidemic in England: estimation using different data sources from changing surveillance practices
Source: BMC Public Health. 2022 Apr 11;22:716. doi: 10.1186/s12889-022-13069-0 (PMC8996221; doi:10.1186/s12889-022-13069-0)
Supplement: Supplementary file 1 — Additional file 1. Supplementary Materials. [file 12889_2022_13069_MOESM1_ESM.docx]

Supplementary Materials

# A Spatial aggregation

Four sub-regions of Buckinghamshire were aggregated in order to match most recent population estimates, since the LTLA was recently sub-divided. The City of London was combined with Westminster due to its very small resident population, and the Isles of Scilly were excluded since no COVID-19-related deaths had been reported there within this time period. Overall, reported deaths were attributed to 312 spatial units across England.

LTLAs can be classified into one of four geographical categories: London borough (10.3 % of total LTLAs), metropolitan district (11.5 %), non-metropolitan district (60.3 %) and unitary authority (17.9 %). The former two categories capture the major urban areas of the country (including Birmingham, Liverpool, Manchester, Sheffield, Leeds and Newcastle) with high connectivity both nationally and internationally, while the latter capture predominantly rural areas and smaller towns or cities.

# B Age-adjusted expected deaths

Specifically, if [
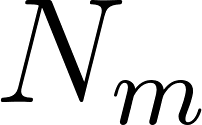
](https://www.codecogs.com/eqnedit.php?latex=N_m#0) is the total observed deaths in age group [
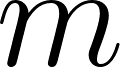
](https://www.codecogs.com/eqnedit.php?latex=m#0) and [
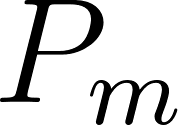
](https://www.codecogs.com/eqnedit.php?latex=P_m#0) the estimated total population of England within the same age group, then [
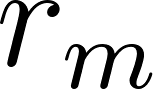
](https://www.codecogs.com/eqnedit.php?latex=r_m#0) is defined as the total age-specific mortality rate

[
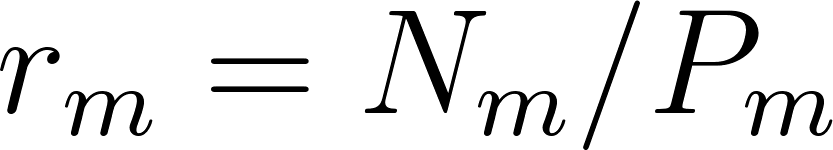
](https://latex-staging.easygenerator.com/eqneditor/editor.php?latex=r_m%3DN_m%2FP_m#0)

over the whole period. These rates are scaled down to estimated average rates per week by dividing by the number of observed weeks in the study period (~ 25). If [
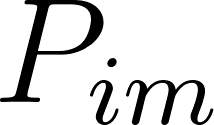
](https://www.codecogs.com/eqnedit.php?latex=P_%7Bim%7D#0) is the estimated population in age group [
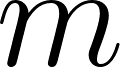
](https://www.codecogs.com/eqnedit.php?latex=m#0) within local authority [
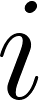
](https://www.codecogs.com/eqnedit.php?latex=i#0), then the expected number of deaths per week, [
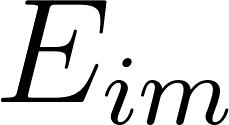
](https://www.codecogs.com/eqnedit.php?latex=E_%7Bim%7D#0), for age group [
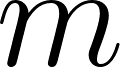
](https://www.codecogs.com/eqnedit.php?latex=m#0) in LA [
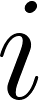
](https://www.codecogs.com/eqnedit.php?latex=i#0) is calculated as

[
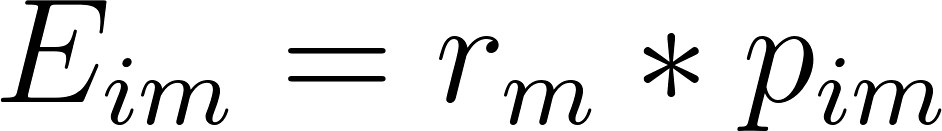
](https://www.codecogs.com/eqnedit.php?latex=E_%7Bim%7D%3Dr_m*p_%7Bim%7D#0)

Finally, the expected deaths overall in LA [
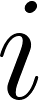
](https://www.codecogs.com/eqnedit.php?latex=i#0) is

[
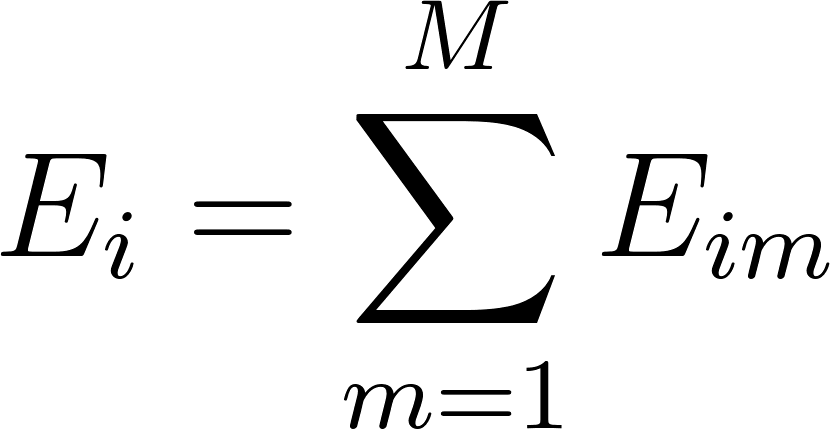
](https://latex-staging.easygenerator.com/eqneditor/editor.php?latex=E_i%20%3D%20%5Csum_%7Bm%3D1%7D%5EM%20E_%7Bim%7D%20#0)

where [
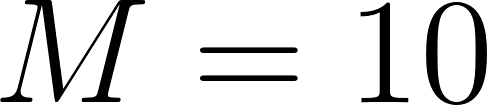
](https://latex-staging.easygenerator.com/eqneditor/editor.php?latex=M%20%3D%2010#0) denotes the total number of age groups. These expected values form a baseline which assumes all LTLAs exhibit the same age-specific mortality rates, and that these rates are constant over the observed period. We then conduct the analysis on the standardised mortality ratio, SMR, of observed deaths, per week and LA, over expected.

# C Model Formulae

The overall structure of fitted models for number of deaths [
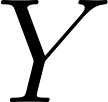
](https://www.codecogs.com/eqnedit.php?latex=Y#0) and expected count [
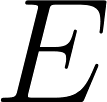
](https://www.codecogs.com/eqnedit.php?latex=E#0) is as follows:

[
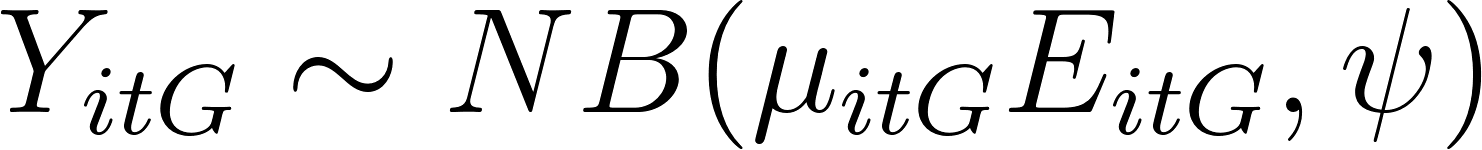
](https://www.codecogs.com/eqnedit.php?latex=%20Y_%7BitG%7D%20%5Csim%20NB(%5Cmu_%7BitG%7D%20E_%7BitG%7D%2C%5Cpsi)#0)

[
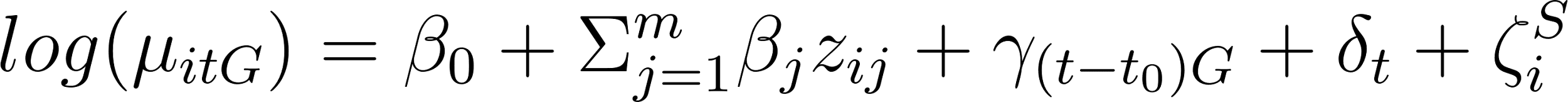
](https://www.codecogs.com/eqnedit.php?latex=%20log(%5Cmu_%7BitG%7D)%20%3D%20%5Cbeta_0%20%2B%20%5CSigma_%7Bj%3D1%7D%5Em%20%5Cbeta_j%20z_%7Bij%7D%20%2B%20%5Cgamma_%7B(t-t_0)G%7D%20%2B%20%5Cdelta_%7Bt%7D%20%2B%20%5Czeta_i%5E%7BS%7D%20#0)

for LTLA [
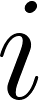
](https://www.codecogs.com/eqnedit.php?latex=i#0) in calendar week [
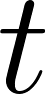
](https://www.codecogs.com/eqnedit.php?latex=t#0), where [
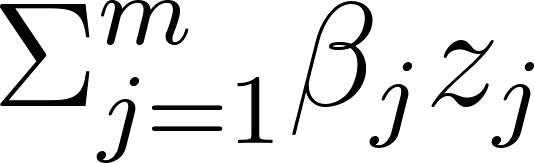
](https://www.codecogs.com/eqnedit.php?latex=%5CSigma_%7Bj%3D1%7D%5Em%20%5Cbeta_j%20z_j#0) denotes the contribution of fixed covariate effects, [
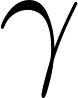
](https://www.codecogs.com/eqnedit.php?latex=%5Cgamma#0) and [
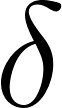
](https://www.codecogs.com/eqnedit.php?latex=%5Cdelta#0) the temporal random effects on epidemic week (denoted
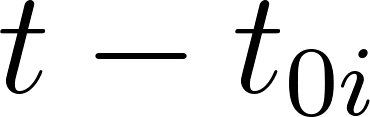
 for the week of first
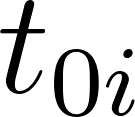
 death) and calendar week respectively, and [
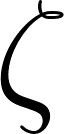
](https://www.codecogs.com/eqnedit.php?latex=%5Czeta#0) the spatial random effect. *NB* reflects the chosen negative binomial likelihood.

The temporal random effects are defined with random walk (RW) correlation structures. A random walk of order one (RW1) assumes that the increments $\delta_{t}-\delta_{t-1}$ between each time step are Gaussian distributed with mean 0 and precision $\tau$. A second order random walk (RW2) assumes the same of the second order increments $\delta_{t}-{2\delta}_{t-1}-\delta_{t-2}$ and hence describes a smoother trend. Specifically, [
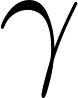
](https://www.codecogs.com/eqnedit.php?latex=%5Cgamma#0) is modelled by a second-order random walk with precision [
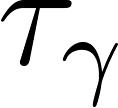
](https://www.codecogs.com/eqnedit.php?latex=%5Ctau_%5Cgamma#0), fit either across all LTLAs or replicated by geography [
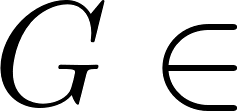
](https://www.codecogs.com/eqnedit.php?latex=G%20%5Cin%20#0) {*London borough*, *metropolitan district*, *non-metropolitan district*, *unitary authority*}. [
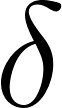
](https://www.codecogs.com/eqnedit.php?latex=%5Cdelta#0) is modelled by a first-order random walk with precision [
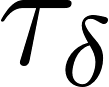
](https://www.codecogs.com/eqnedit.php?latex=%5Ctau_%5Cdelta#0). [
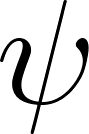
](https://www.codecogs.com/eqnedit.php?latex=%5Cpsi#0) is the size parameter (1/overdispersion) for the negative binomial distribution.

Three candidate structures for the spatial random effect were considered. The index [
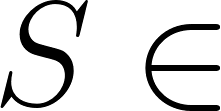
](https://www.codecogs.com/eqnedit.php?latex=S%20%5Cin%20#0) {*Null*, IID, *BYM*} indicates either no spatial model, the completely unstructured IID model or the Besag-York-Mollie spatially-structured model parameterised with precision [
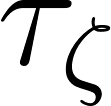
](https://www.codecogs.com/eqnedit.php?latex=%5Ctau_%5Czeta#0) and mixing parameter [
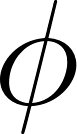
](https://www.codecogs.com/eqnedit.php?latex=%5Cphi#0). These are defined as follows:

Null:

[
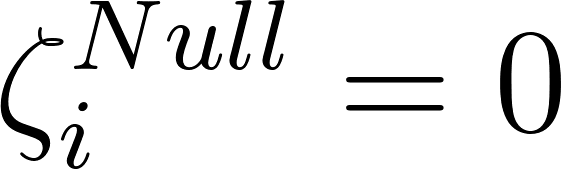
](https://www.codecogs.com/eqnedit.php?latex=%5Czeta_i%5E%7BNull%7D%20%3D%200#0)

IID:

[
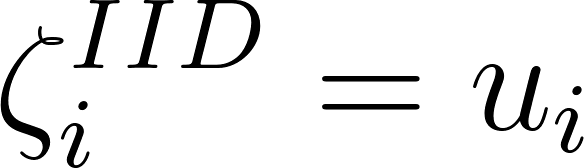
](https://www.codecogs.com/eqnedit.php?latex=%5Czeta_i%5E%7BIID%7D%20%3D%20u_i#0)

BYM:

[
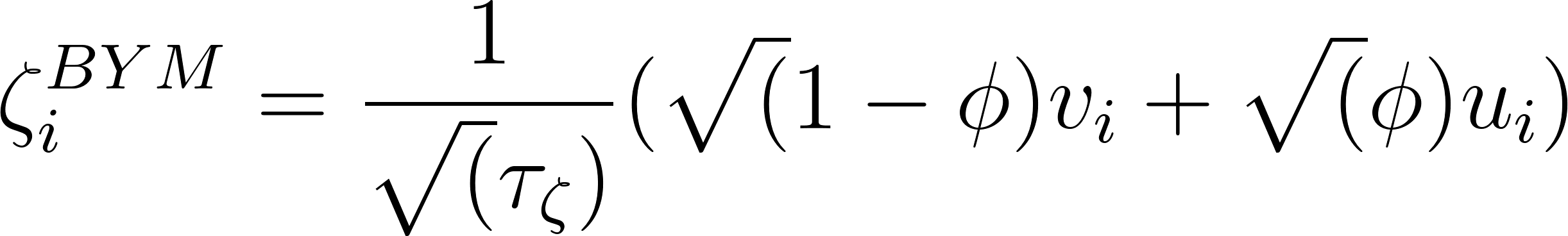
](https://www.codecogs.com/eqnedit.php?latex=%5Czeta_i%5E%7BBYM%7D%20%3D%20%20%5Cfrac%7B1%7D%7B%5Csqrt(%5Ctau_%5Czeta)%7D(%5Csqrt(1-%5Cphi)v_i%20%2B%20%5Csqrt(%5Cphi)u_i)#0)

## Priors

Gaussian priors with mean 0 and precision 0.1 were specified for the fixed covariate effects.

Penalised complexity priors were specified for the precisions of the three structured random effects (temporal and spatial) such that [
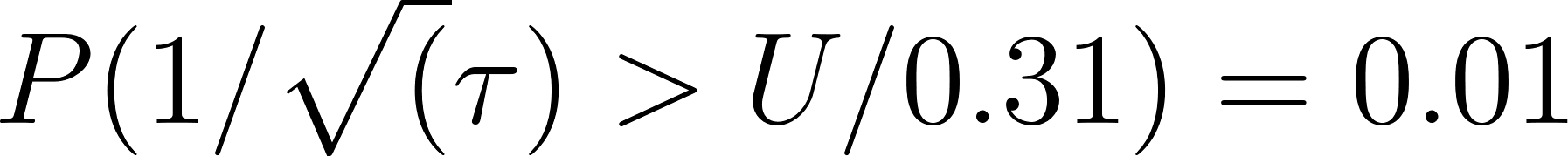
](https://www.codecogs.com/eqnedit.php?latex=P(1%2F%5Csqrt(%5Ctau)%20%3E%20U%2F0.31)%20%3D%200.01%20#0), with the upper limit [
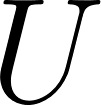
](https://www.codecogs.com/eqnedit.php?latex=U#0) defined as the standard deviation of residuals from the null, fixed-effect-only model, averaged over the relevant index (epidemic week, calendar week, LTLA). The BYM mixing parameter [
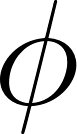
](https://www.codecogs.com/eqnedit.php?latex=%5Cphi#0) is also given a penalised complexity prior, such that [
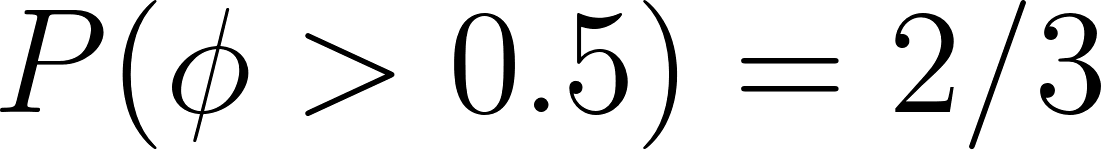
](https://www.codecogs.com/eqnedit.php?latex=P(%5Cphi%20%3E%200.5)%20%3D%202%2F3#0).

# D Rate of detection under symptomatic community testing

The ONS COVID-19 infection survey was piloted from April 2020, conducting PCR tests in samples of the population in order to estimate the prevalence of test-positive infections in the country over time. Estimates during this early period are presented as a percentage of the population who would test PCR-positive, by rolling fortnight, and were translated to an approximate weekly incidence by dividing by two, assuming test-positivity duration of one week and simple steady-state dynamics.

Assuming the population of England to be 56 million, the total weekly incidence of test-positives was calculated for weeks starting 18 May to 15 June 2020. The cumulative count of infections over this period was then compared to the cumulative count of confirmed cases to estimate the detection rate of infections under expanded surveillance.

# Supplementary Figures


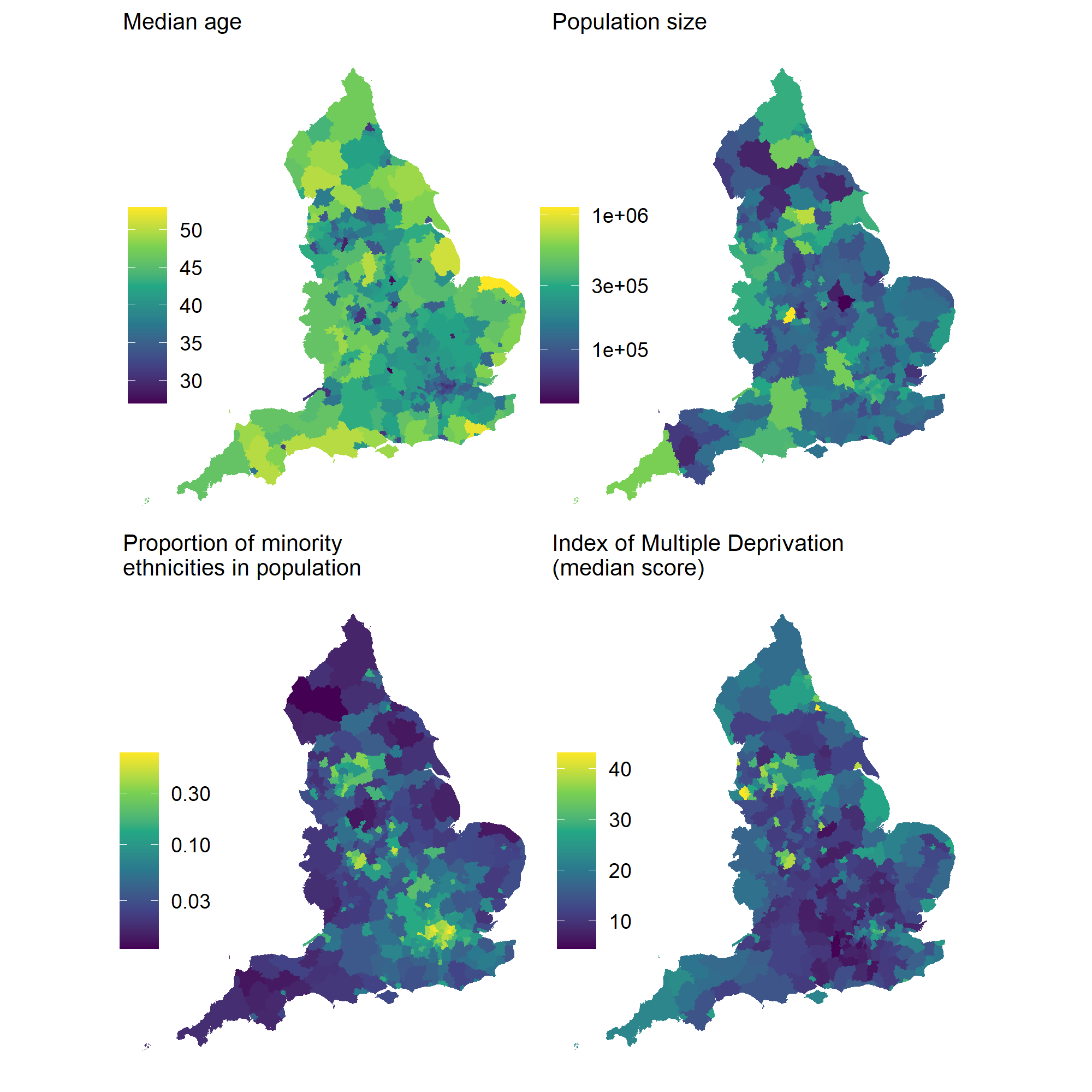


**Figure S1: Distribution of LTLA-level characteristics used in modelling of mortality risk.** Younger age and greater minority proportion are characteristic of urban centres, whereas deprivation is more pronounced across northern LTLAs.


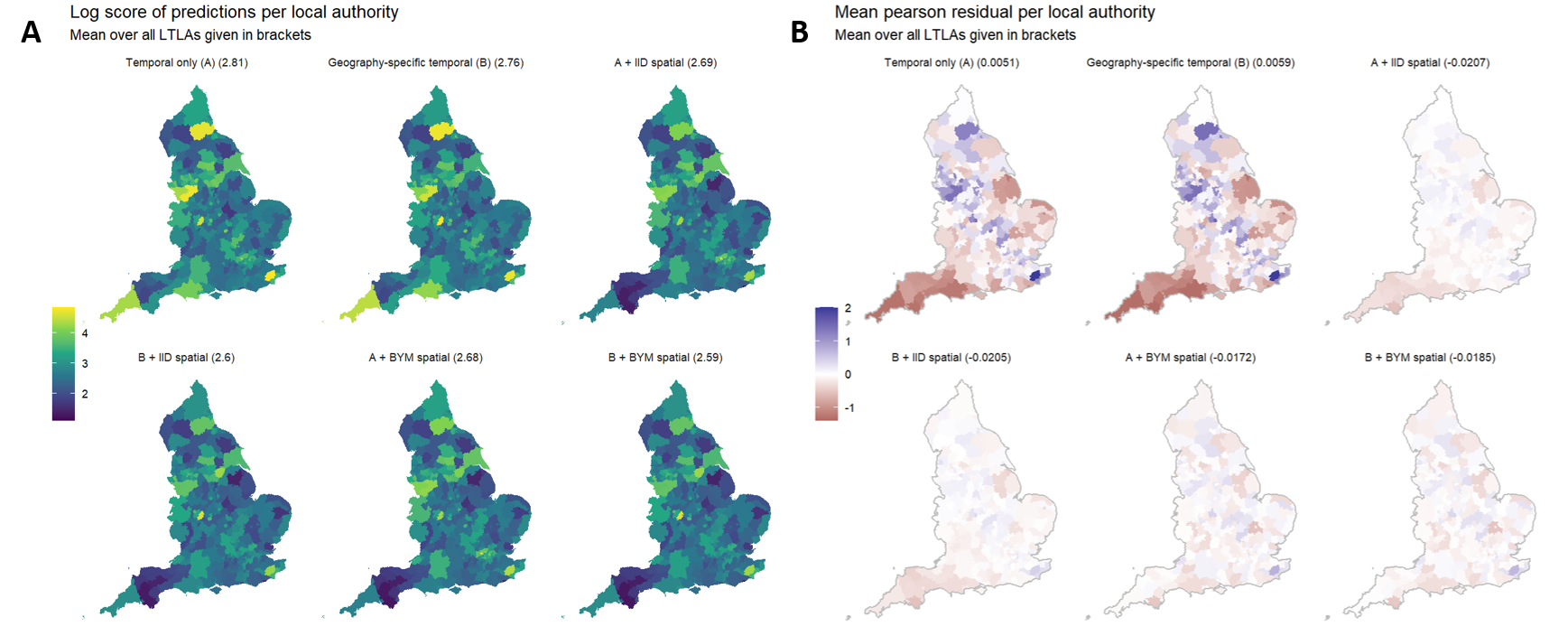
**Figure S2: Averaged log scores (top) and Pearson residuals (bottom) from models fitted to weekly deaths per local authority which occurred between 2020-01-01 and 2020-06-30.** Adding spatial random effects reduces the magnitude of error overall, with the conditional autoregressive structure from the BYM model providing the best cross-validated fit.

*
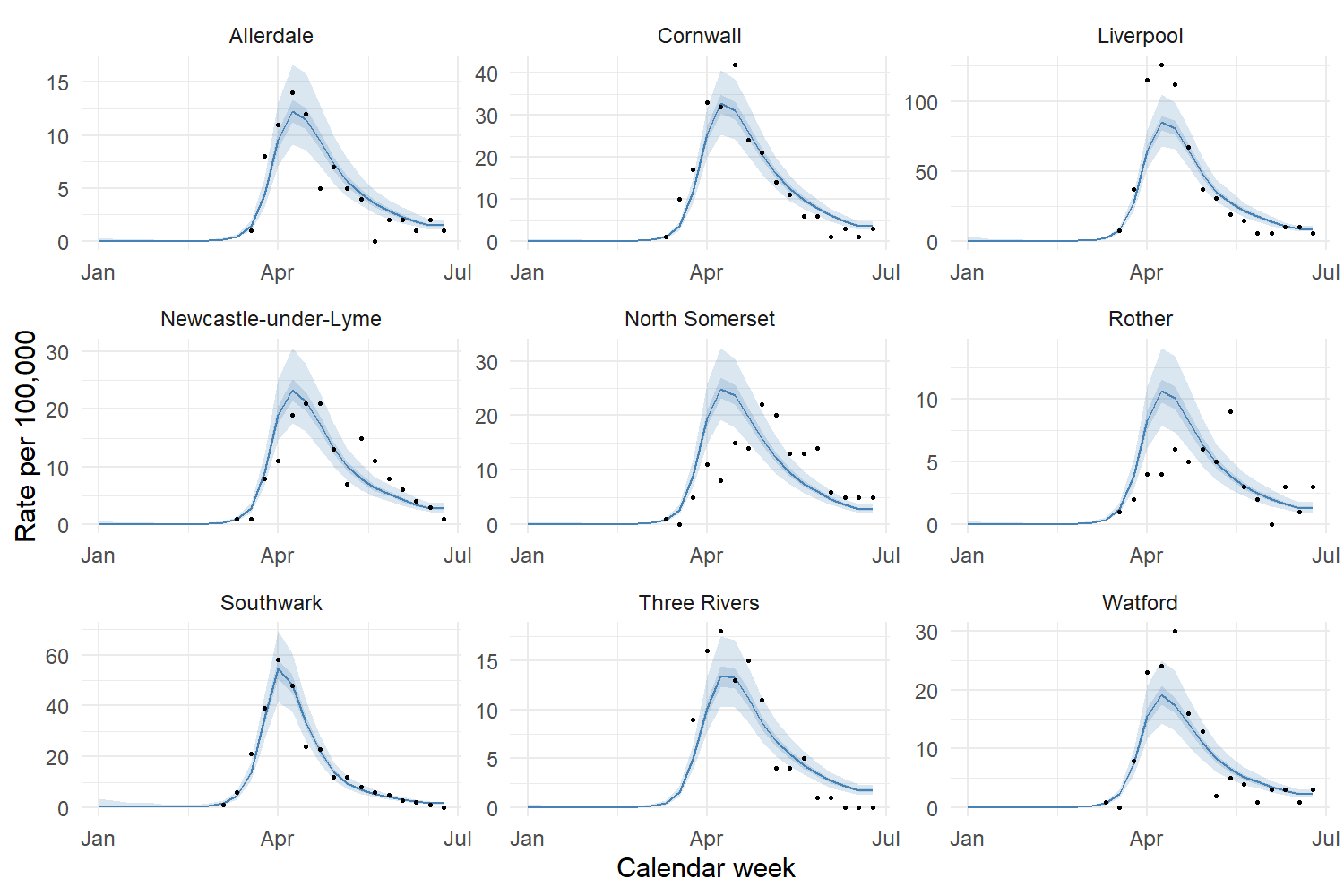
*

**Figure S3: Fit of the selected model for nine randomly sampled LTLAs, over 1,000 posterior samples.** For the LTLA fits, observed rates of COVID-19-related death per 100,000 are shown in black, with 50-98% credible intervals.


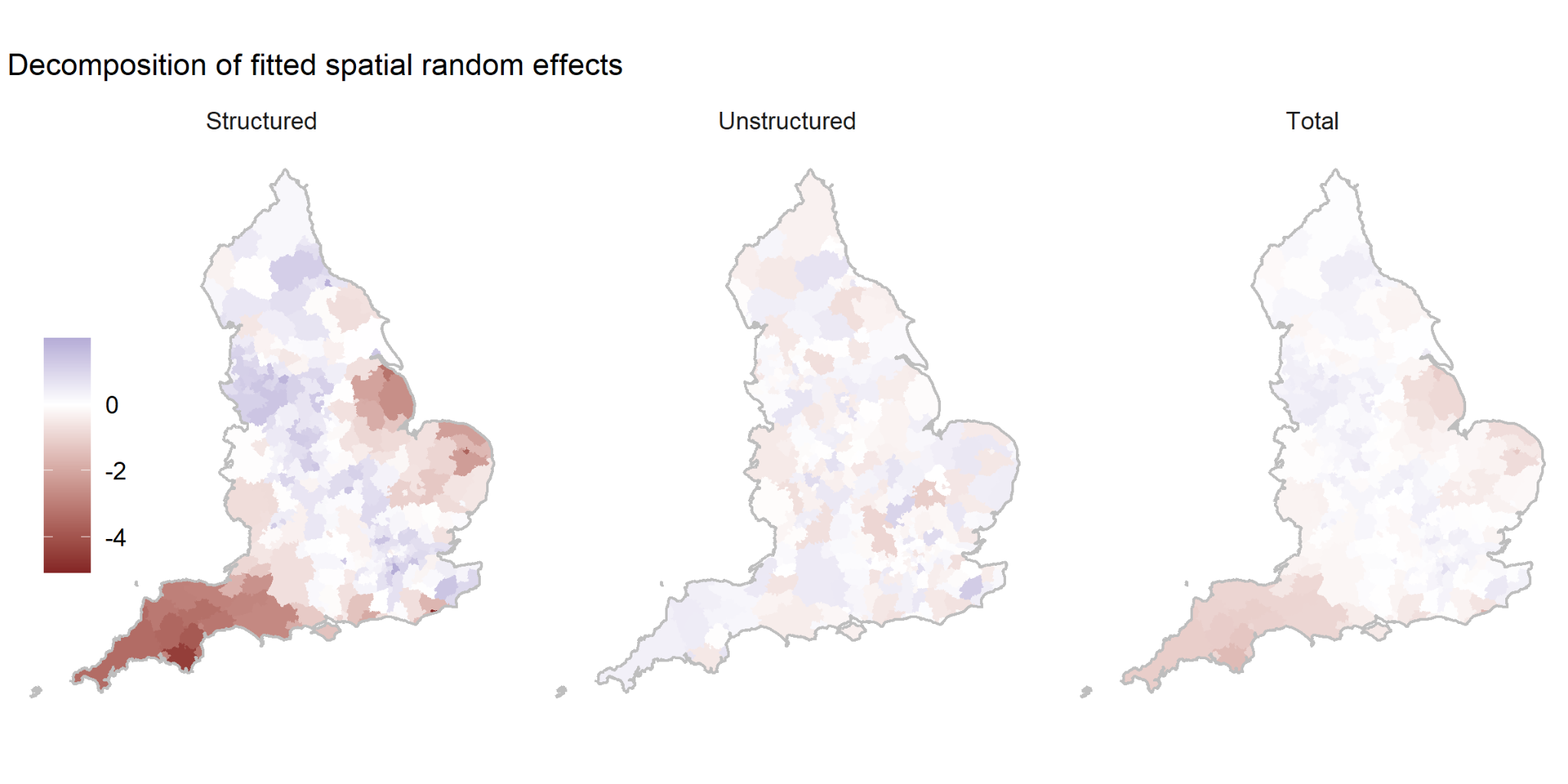
**Figure S4: Decomposition of the fitted BYM spatial model into structured and unstructured components.** For the selected model, the percentage of residual spatial variation attributable to the local correlation structure was estimated as 95% (95% CrI [86 - 99]).


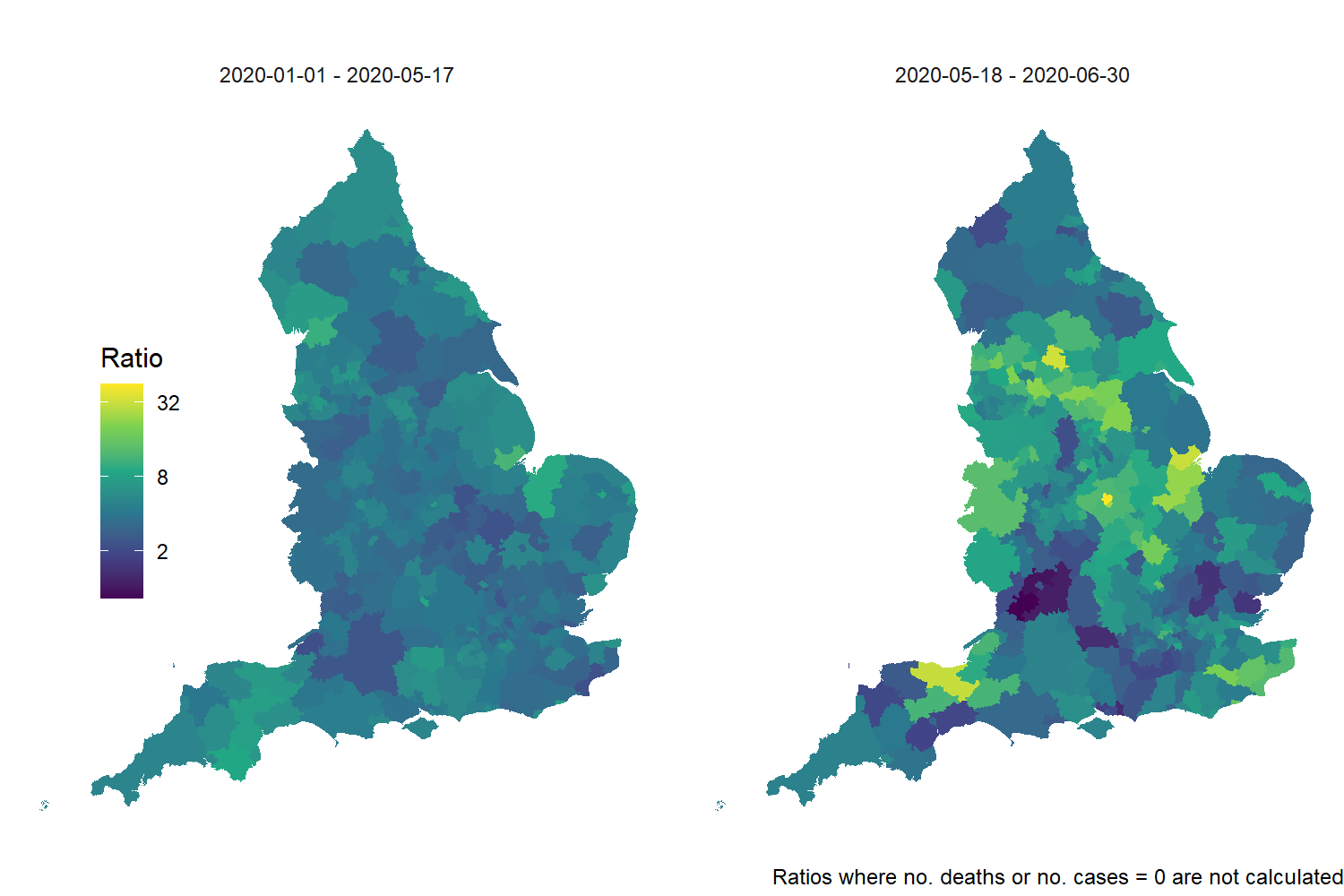


**Figure S5: Median ratio between weekly observed cases and one-week-lagged modelled deaths per LTLA (population case-fatality ratio), before (left) and after (right) expansion of pillar 2 testing for all symptomatic individuals from 2020-05-18.** Greater variation post-P2 expansion will in part be attributable to overall smaller counts of deaths per LTLA.

**
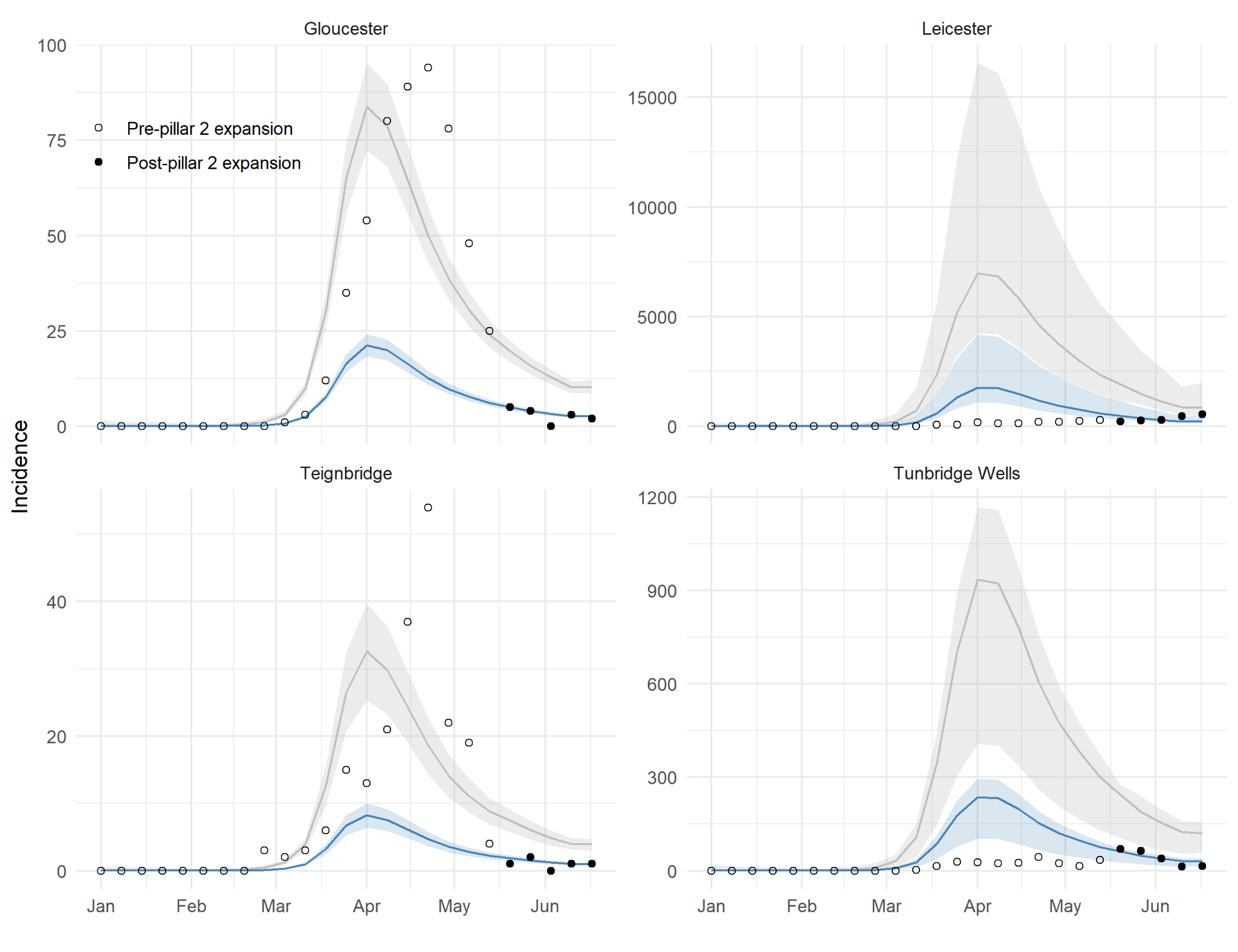
Figure S6: Predicted-P1+P2 cases (blue) and total infections (grey) over time, within LTLAs with the highest and lowest estimated detection rates.** Estimates for Gloucester and Teignbridge were 96.6% [87%, 110%] and 96.1% [81%, 121%], while for Leicester and Tunbridge Wells were 6.7% [3%, 11%] and 6.8% [6%, 15%].

# Supplementary Tables

**Table S1: Summary of LTLA-level characteristics, overall and by geography type (median [IQR])**. Age is defined as the estimated median according to age-specific population estimates for each LTLA, IMD as the median score across lower super output areas (the level at which the score is calculated) within each LTLA, and % minority population as the percentage of the LTLA population identifying as non-white according to the most recent census (2011).

|  | | **Median age** | **Median IMD score** | **% Minority population** |
| --- | --- | --- | --- | --- |
| Overall | | 41 [37, 45] | 16.1 [11.4, 22.4] | 0.05 [0.03, 0.13] |
| By geography | London Borough | 34.5 [33, 36] | 20.4 [13.9, 26.5] | 0.39 [0.31, 0.47] |
|  | Metropolitan District | 39 [35, 41] | 27.2 [21.4, 31] | 0.11 [0.04, 0.19] |
|  | Non-metropolitan District | 43 [40, 46] | 13.8 [10.8, 18.4] | 0.04 [0.02, 0.07] |
|  | Unitary Authority | 39.5 [35.75, 43] | 19.1 [13, 23.9] | 0.06 [0.03, 0.14] |

**Table S2: Estimated coefficients for LTLA-level covariates (posterior mean and 95% credible interval), from the final selected model.** Estimates are multiplicative due to the log link function, hence a value greater than one would indicate a positive association with COVID-19-related mortality rate and a value less than one a negative effect. A higher percentage of minority ethnicities and higher deprivation quintile in the LTLA were found to be associated with higher rates of COVID-19-related mortality, after accounting for the age and size of the population. Fixed effect estimates (posterior mean and 95% credible interval) from the final selected model.

| **Covariate** | | **Estimate [95% CrI]** |
| --- | --- | --- |
| % minority ethnicity | | 1.01 [1.006, 1.015] |
| IMD score quintile | 1 (least deprived) | 1 |
|  | 2 | 1.03 [0.96, 1.12] |
|  | 3 | 1.17 [1.06, 1.30] |
|  | 4 | 1.27 [1.10, 1.47] |
|  | 5 (most deprived) | 1.21 [0.97, 1.49] |

**Table S3: Sensitivity analysis comparing predicted-P1+P2 cases under assumed lags of two and three weeks between confirmatory testing and death.** As in Table 2, counts reflect the hypothetical scenario in which expanded surveillance (hospital- and community-based symptomatic testing) were available from the start of the epidemic. The differences explored here are a result of assuming a longer (either two or three week) average lag between the date a case is initially swabbed for testing and the date of death.

|  | **Observed, test- confirmed cases**  **(*up to week starting 2020-06-10*)** | **Two week lag** | | **Observed, test- confirmed cases**  **(*up to week starting 2020-06-03*)** | **Three week lag** | |
| --- | --- | --- | --- | --- | --- | --- |
|  |  | **Predicted**  **(*median [IQR]*)** | **Percentage**  **difference** |  | **Predicted**  **(*median [IQR]*)** | **Percentage**  **difference** |
| England total | 226,522 | 418,627 [352,699 - 493,737] | 84.8 [55.7 - 118] | 220,218 | 515,598 [452,200 - 582,182] | 134.1 [105.3 - 164.4] |
|  | | | | | | |
| London Borough | 33,118 | 54,447 [45,905 - 63,585] | 64.4 [38.6 - 92] | 32,809 | 67,350 [60,818 - 75,379] | 105.3 [85.4 - 129.8] |
| Metropolitan District | 61,976 | 130,758 [117,755 - 144,591] | 111 [90 - 133.3] | 59,757 | 153,856 [141,322 - 165,896] | 157.5 [136.5 - 177.6] |
| Non-  metropolitan District | 77,965 | 125,167 [101,393 - 151,902] | 60.5 [30 - 94.8] | 76,100 | 159,341 [133,909 - 185,609] | 109.4 [76 - 143.9] |
| Unitary Authority | 53,463 | 107,980 [91,151 - 127,351] | 102 [70.5 - 138.2] | 51,552 | 134,568 [120,599 - 149,287] | 161 [133.9 - 189.6] |

**Table S4: Summary of observed confirmed cases and estimated total infections, by geography and region.**

|  | Observed,  test-confirmed cases  (up to week starting  2020-06-17) | Estimated total infections  (median [98% CrI]) | Percentage  difference |
| --- | --- | --- | --- |
| England | 231,817 | 1,323,622 [1,038,213 – 1,737,564] | 17.5 [13.3 - 22.3] |
|  | | | |
| London Borough | 33,399 | 172,478 [122,976 – 233,570] | 19.4 [14.3 - 27.2] |
| Metropolitan District | 64,007 | 433,397 [345,610 – 568,269] | 14.8 [11.3 - 18.5] |
| Non-metropolitan  District | 79,441 | 386,269 [292,924 – 511,017] | 20.6 [15.5 - 27.1] |
| Unitary Authority | 54,970 | 330,717 [247,024 – 479,398] | 16.6 [11.5 - 22.3] |
|  | | | |
| East Midlands | 20,053 | 176,864 [131,958 – 271,843] | 11.3 [7.4 - 15.2] |
| East of England | 23,058 | 116,037 [88,180 – 151,430] | 19.9 [15.2 - 26.1] |
| London | 33,399 | 172,478 [122,976 – 233,570] | 19.4 [14.3 - 27.2] |
| North East | 14,981 | 48,207 [36,572 – 64,796] | 31.1 [23.1 - 41] |
| North West | 41,607 | 258,741 [212,280 – 315,380] | 16.1 [13.2 - 19.6] |
| South East | 33,249 | 138,331 [106,817 – 184,415] | 24 [18 - 31.1] |
| South West | 12,623 | 47,713 [27,010 – 65,821] | 26.5 [19.2 - 46.7] |
| West Midlands | 24,874 | 129,636 [104,508 – 156,690] | 19.2 [15.9 - 23.8] |
| Yorkshire and  The Humber | 27,973 | 235,422 [184,770 – 335,078] | 11.9 [8.3 - 15.1] |
