# Supplementary figures and images for: The local burden of disease during the first wave of the COVID-19 epidemic in England: estimation using different data sources from changing surveillance practices
Source: BMC Public Health. 2022 Apr 11;22:716. doi: 10.1186/s12889-022-13069-0 (PMC8996221; doi:10.1186/s12889-022-13069-0)

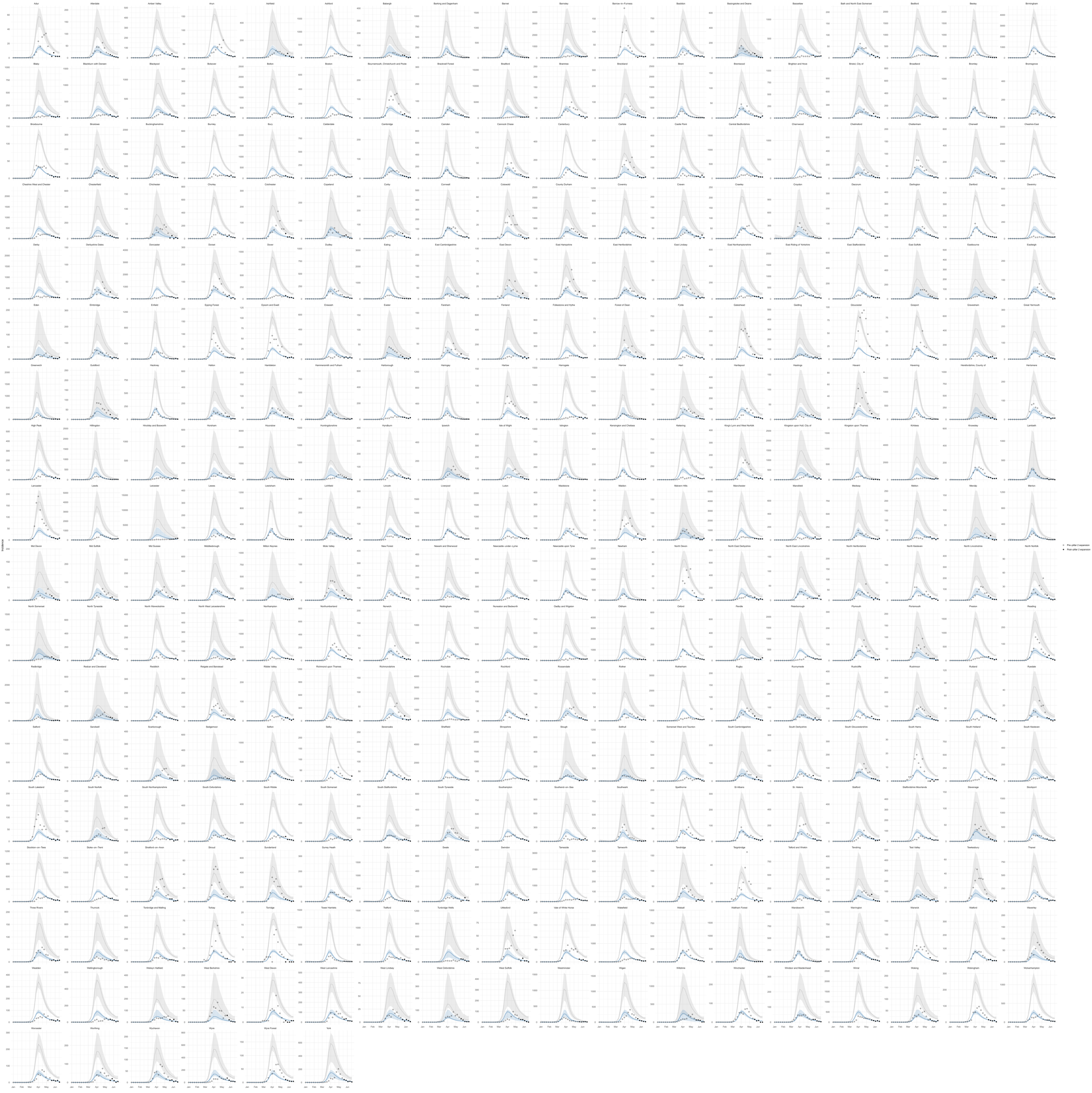

Supplement: Supplementary file 2 — Additional file 2. Final predicted-P1+P2 and total infections for the entire time series in each LTLA. [file 12889_2022_13069_MOESM2_ESM.pdf]
